# ARL4C might serve as a prognostic factor and a novel therapeutic target for gastric cancer: bioinformatics analyses and biological experiments

**DOI:** 10.1111/jcmm.16366

**Published:** 2021-03-16

**Authors:** Ning Xie, Yunfan Bai, Lu Qiao, Yuru Bai, Jian Wu, Yan Li, Mingzuo Jiang, Bing Xu, Zhen Ni, Ting Yuan, Yongquan Shi, Kaichun Wu, Feng Xu, Jinhai Wang, Lei Dong, Na Liu

**Affiliations:** ^1^ Department of Gastroenterology the Second Affiliated Hospital of Xi’an Jiaotong University Xi'an China; ^2^ Shaanxi Key Laboratory of Gastrointestinal Motility Disorders Xi’an Jiaotong University Xi'an China; ^3^ Department of Computational Biology and Medical Sciences Graduate School of Frontier Sciences University of Tokyo Kashiwa Japan; ^4^ State Key Laboratory of Cancer Biology National Clinical Research Center for Digestive Diseases and Xijing Hospital of Digestive Diseases the Fourth Military Medical University Xi'an China; ^5^ The Key Laboratory of Biomedical Information Engineering of Ministry of Education School of Life Science and Technology Xi’an Jiaotong University Xi’an China; ^6^ Bioinspired Engineering and Biomechanics Center (BEBC) Xi’an Jiaotong University Xi’an China

**Keywords:** ARL4C, ARLs, clinical values, gastric cancer, TGF‐β1

## Abstract

The ADP‐ribosylation factor‐like proteins (ARLs) have been proved to regulate the malignant phenotypes of several cancers. However, the exact role of ARLs in gastric cancer (GC) remains elusive. In this study, we systematically investigate the expression status, interactive relations, potential pathways, genetic variations and clinical values of ARLs in GC. We find that ARLs are significantly dysregulated in GC and involved in various cancer‐related pathways. Subsequently, machine learning models identify ARL4C as one of the two most significant clinical indicators among ARLs for GC. Furthermore, ARL4C silencing remarkably inhibits the growth and metastasis of GC cells both i*n vitro* and in vivo. Moreover, enrichment analysis indicates that ARL4C is highly correlated with TGF‐β1 signalling. Correspondingly, TGF‐β1 treatment dramatically increases ARL4C expression and ARL4C knockdown inhibits the phosphorylation level of Smads, downstream factors of TGF‐β1. Meanwhile, the coexpression of ARL4C and TGF‐β1 worsens the prognosis of GC patients. Our work comprehensively demonstrates the crucial role of ARLs in the carcinogenesis of GC and the specific mechanisms underlying the GC‐promoting effects of TGF‐β1. More importantly, we uncover the great promise of ARL4C‐targeted therapy in improving the efficacy of TGF‐β1 inhibitors for GC patients.

## 1 INTRODUCTION

Gastric cancer (GC) is the fifth most common malignancy and the third leading cause of cancer‐related deaths worldwide.^1^ GC is generally diagnosed at the advanced stages because early GC is commonly asymptomatic or mild, leading to unsatisfactory prognosis.^2^ Thus, it is critical to further discover the molecular mechanisms underlying the tumorigenesis and progression of GC, and to identify novel promising biomarkers with higher sensitivity and specificity for early detection and prognosis evaluation.

The ADP‐ribosylation factor (ARF) family members of small GTP‐binding (G) proteins, comprising the ARFs, tripartite motif‐containing protein 23 (TRIM23), ARF‐like (ARL) proteins and SAR1, belong to the Ras superfamily.^3^ ARLs, which are structurally similar to ARF family members, have been identified as the key regulators that control vesicular transport, membrane traffic, organelle structure, cytoskeleton organization and cell migration through cyclic regulation between their GTP‐bound active state and their GDP‐bound inactive state.^3^, ^4^, ^5^ For instance, deletion of ARL13B in the distal nephron at the perinatal stage damages cilia biogenesis and leads to rapid kidney cyst.^6^ ARL4A could alter the integrity of the Golgi structure^7^ and modulate cell motility by regulating Cdc42 activity.^8^ Recently, accumulating evidence suggests that the aberrant regulation of ARLs plays a critical role in tumorigenesis of several cancers.^9^, ^10^ For example, ARL2 overexpression inhibits the malignant phenotypes of glioma cells and predicts better clinical outcomes of glioma patients,^11^ while its down‐regulation could suppress the invasion and growth of cervical cancer cells.^12^ ARL4C expression promotes the progression of colorectal and lung cancers in vitro and in vivo.^13^, ^14^ In addition, it has been identified as a peritoneal dissemination (PD)–associated gene for GC.^14^ ARL8B is essential for the 3D invasive growth of prostate cancer both in vitro and in vivo.^15^ A recent study reveals that ARL13B can enhance the migration and invasion of breast cancer cells via controlling integrin‐mediated signalling pathway.^16^ Meanwhile, ARL13B would act as a negative prognostic indicator for GC patients and promote GC progression via regulating Smo trafficking and activating the Hedgehog signalling pathway.^17^ However, the clinical values and biological functions of ARLs in GC remain elusive.

In this study, we firstly comprehensively investigate the expression profiles, hallmark pathways, genetic alterations and clinical values of ARLs in GC. We find that ARL4C and ARL13B are the two most important diagnostic and prognostic indicators among ARLs for GC by machine learning models. Further in vitro and in vivo experiments demonstrate that down‐regulation of ARL4C dramatically inhibits tumorigenesis and metastasis of GC cells. More importantly, we discover that ARL4C is highly related to TGF‐β1 signalling pathway, which is further confirmed by our discovery that the coexpression of ARL4C and TGF‐β1 predicts worse survival for GC patients than ARL4C or TGF‐β1, respectively.

## 2 METHODS AND MATERIALS

### 2.1 Online bioinformatics analyses

The differential expression between gastric cancer and adjacent normal tissues from TCGA and GTEx data sets was displayed by GEPIA (http://www.gepia.cancer‐pku.cn/detail.php) online tool.^18^ The Oncomine database (http://www.oncomine.org) was utilized to analyse the mRNA expression levels of ARLs in digestive system cancer.^19^ Gene‐gene networks and functions of ARLs were evaluated by GeneMANIA online tool (http://www.genemania.org).^20^ Genomic alterations of ARLs in GC available at the TCGA database (TCGA, Firehose Legacy) were assessed using the cBioPortal online tool (http://www.cbioportal.org).^21^ DNA methylation status of ARL4C was analysed using MEXPRESS (https://www.mexpress.be/).^22^ The Kaplan‐Meier plotter database (http://www.kmplot.com/analysis/) was used to investigate the predictive effects of ARLs on the overall survival of GC patients.^23^ All the analyses were performed according to the guidelines of these databases.

### 2.2 Gene set variation analysis (GSVA)

GSVA, a functional enrichment software, was utilized to estimate the variation of pathway activity over a sample population in an unsupervised manner.^24^ Hallmark gene sets and Kyoto Encyclopedia of Genes and Genomes (KEGG) pathway sets of ARLs in GC were carried out by 'ggplot2' R package based on GSVA software. *P* < 0.05 and FDR < 0.05 were set as the screening standard.

### 2.3 Logistic regression model construction and validation

TCGA and GTEx data sets were obtained from UCSC database (https://www.genome.ucsc.edu) to perform the analysis of ARLs’ diagnostic values on GC patients. The patients were randomly administered into a training (75%) and a validation cohort (25%). Logistic regression was carried out to identify the diagnostic biomarkers with statistical significance in the training cohort using the 'glm' function in R platform. Moreover, we evaluated the ability of predicted diagnostic markers in differentiating the GC patients in the validation cohort.

### 2.4 Least absolute shrinkage and selection operator (LASSO) Cox regression analysis

GSE15459 cohort (tumour, n = 200) in the Gene Expression Omnibus (GEO) was utilized to perform the prognostic analyses of ARLs in GC patients (https://www.ncbi.nlm.nih.gov/geo/query/acc.cgi?acc=GSE34942). LASSO Cox regression analysis was conducted using the 'glmnet R package. Tuning parameter (λ) selection in the LASSO model used 10‐fold cross‐validation via minimum criteria. A λ value of 0.0379 was chosen (λ.min) according to 10‐fold cross‐validation.

### 2.5 Enrichment analysis

Pearson's correlation coefficient exceeding 0.5 indicated a good correlation between ARL4C and its coexpressed genes. We employed the 'clusterProfiler' R package for Gene Ontology (GO) and KEGG enrichment analysis of genes coexpressed with ARL4C.

### 2.6 Kaplan‐Meier analysis

We conducted the Kaplan‐Meier analysis to validate the effects of TGF‐β1 and ARL4C on the overall survival of GC patients by R platform.

### 2.7 Nomogram construction and validation

Nomogram was built based on a multivariate Cox analysis using the 'rms' R package. The predictive performance of the nomogram was then validated by decision curve analysis (DCA) and calibration curves.

### 2.8 Clinical samples

The GC tissue microarray (ST‐1503) was purchased from Xi'an Alena Bio. In addition, 12 paired samples of primary GC and adjacent normal tissues were obtained from patients who had undergone GC surgery at Xijing Hospital of Digestive Diseases. All samples were clinically and pathologically verified. The detailed information is shown in Tables S7‐8.

### 2.9 Statistical analysis

Student's paired t test was used to determine statistical significance of differences between two groups. Each experiment was carried out at least three times. Statistical analyses were conducted using the SPSS software (version 21.0). Images were obtained using the GraphPad Prism software (version 7.0). The counting data were represented by frequency or percentage, and the measurement data were expressed as the mean ± standard deviation (SD). *P* < 0.05 was considered statistically significant.

Detailed information of the aforementioned materials and methods and other methods including cell culture and transfection, immunohistochemistry (IHC) and immunofluorescence (IF), Western blot analysis, cell proliferation, colony formation assay and xenograft assay was described in Supporting information.

## 3 RESULTS

### 3.1 The expression profiles and potential signalling pathways of ARLs in GC

**FIGURE 1:**
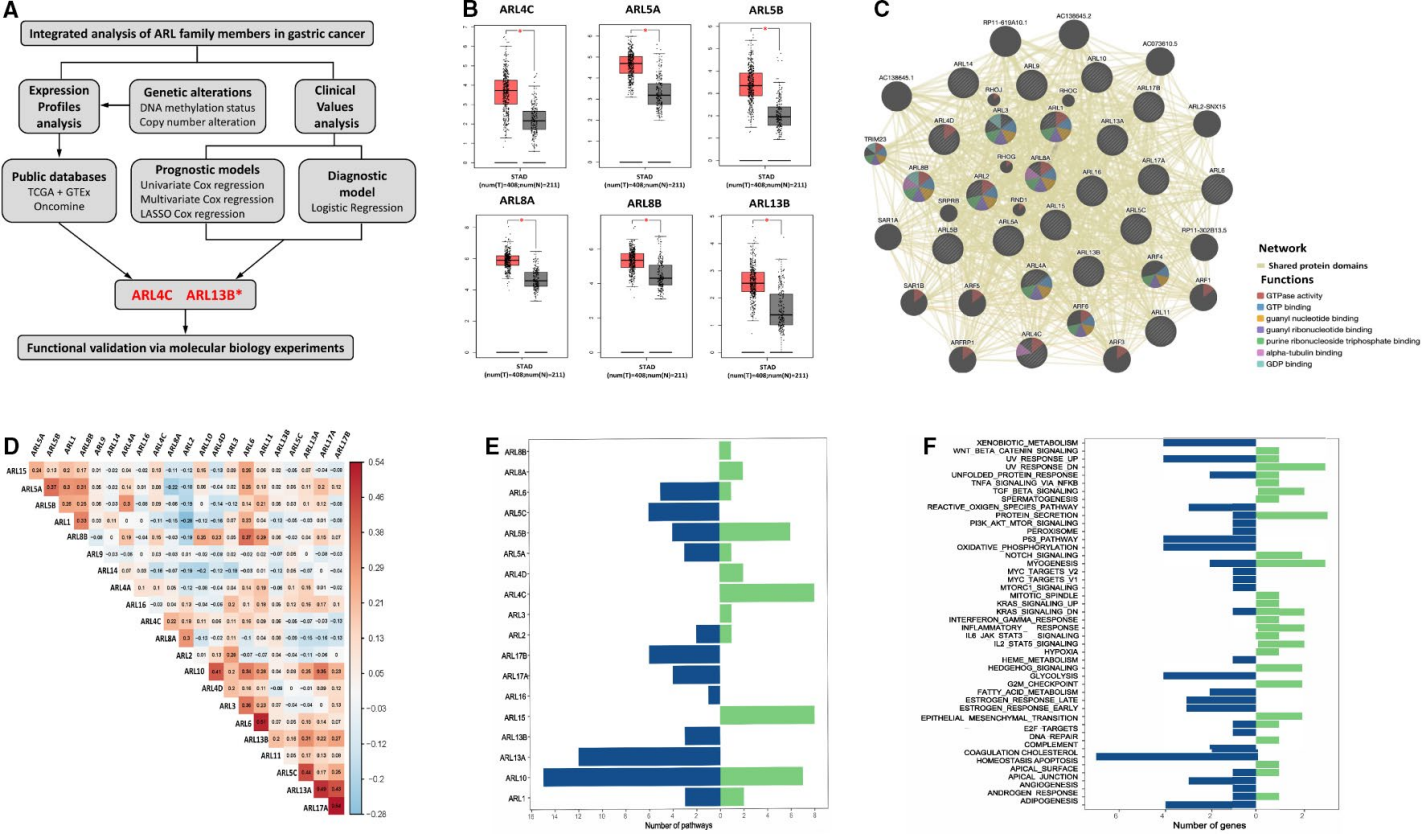
Expression profiles and pathway analysis of ARLs in GC. A, Schematic diagram of research flow. B, Box plots showed the differential expression of ARLs (ARL4C, ARL5A, ARL5B, ARL8A, ARL8B and ARL13B) between GC (red) and adjacent normal tissues (grey) from TCGA by GEPIA (*P < 0.05). C, Gene‐gene interaction network among ARLs. Each node represents a gene. The inter‐node connection lines represented the network types, and the node colours represented the possible functions of respective genes. D, The heat map showed Pearson's correlation values among ARLs in TCGA data set. E, The bar diagram showed the number of pathways regulated by ARLs explored by GSVA. F, The bar diagram displayed the biological processes and signalling pathways regulated by the certain number of ARLs

As shown in Figure 1A, we firstly investigated the expression differences of ARLs between GC tissues and adjacent normal tissues using TCGA, GTEx and Oncomine databases. We found that six ARLs (*ie* ARL4C, ARL5A, ARL5B, ARL8A, ARL8B and ARL13B) were obviously up‐regulated in GC tissues compared with adjacent normal tissues in TCGA and GTEx data sets (*P* < 0.01) (Figure 1B). Meanwhile, six ARLs (*ie* ARL2, ARL4C, ARL10, ARL11, ARL13B and ARL14) were aberrantly regulated in GC patients based on Oncomine (Figure S1A). Both in TCGA and Oncomine databases, ARL4C and ARL13B were significantly up‐regulated in tumour tissues (Figure S1B).

As small GTPase proteins could commonly work synergistically as function hubs to regulate cell biological functions,^25^, ^26^ we conducted a network analysis of ARLs using the GeneMANIA tool, and found a large number of shared protein domains among ARLs (Figure 1C). Based on these findings, we inferred that ARLs were functionally similar genes.

We further performed the correlation analysis of ARLs in GC using TCGA database. As shown in Figure 1D and Table S1, significant correlations were discovered among these genes. To explore the potential oncogenic pathways that ARLs were involved in, we analysed the correlation between the expression of ARLs and the activity of hallmark‐related pathways using GSVA. As shown in Figure 1E,F, various hallmark pathways of cancer were significantly associated with the expression of ARLs, including cholesterol homeostasis (7/22), myogenesis (5/22), UV response up (5/22) and p53 pathway (5/22). Meanwhile, the expression of ARL10 (n = 22), ARL13A (n = 12), ARL5B (n = 10), ARL15 (n = 8) and ARL4C (n = 8) was correlated with a higher number of pathways (Table S2).

### 3.2 Genetic alteration analysis of ARLs in GC

**FIGURE 2:**
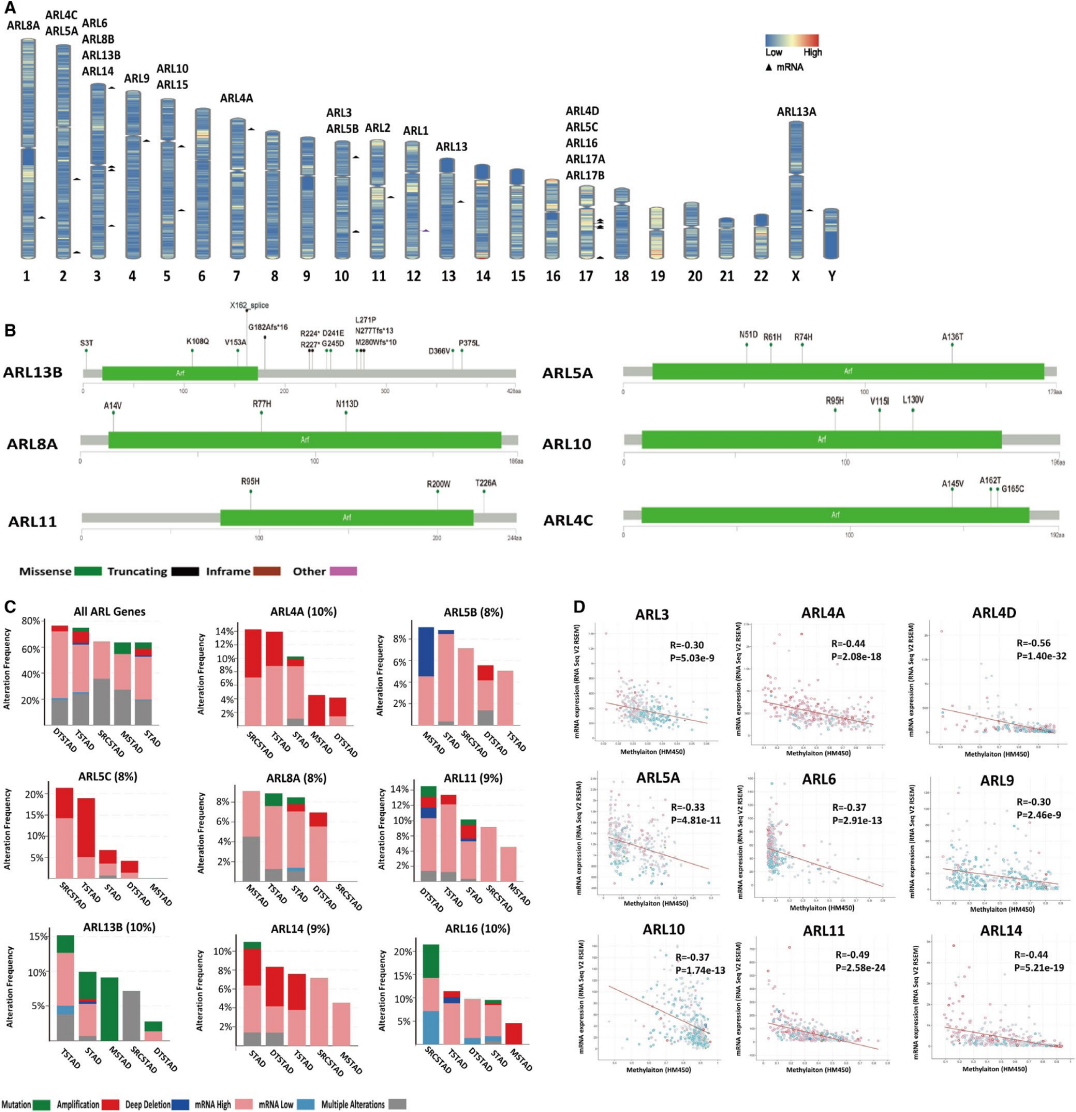
Genetic alterations of ARLs in GC. A, The genomic locations for ARLs (GRCh38/hg38). B, Mutation information of ARL proteins in GC. C, Alteration frequency of ARLs in different GC subtypes including mutations, amplification, deep deletion, mRNA dysregulation and multiple changes. SRCSTAD: Signet ring cell carcinoma of the stomach; TSTAD: tubular stomach adenocarcinoma; STAD: stomach adenocarcinoma; DTSTAD: diffuse‐type stomach adenocarcinoma; MSTAD: mucinous stomach adenocarcinoma. D, Association of ARLs mRNA expression with DNA methylation

To comprehensively understand the expression profiles of ARLs in GC, we analysed the genetic alteration of ARLs in GC. The chromosome status (GRCh38/hg38) shown in Figure 2A and Table S3 clearly displayed the genomic locations of 22 ARLs, and we found that ARLs were unevenly distributed on different chromosomes. Furthermore, we conducted the exact genetic analysis using cBioPortal for Cancer Genomic. From the changes in protein structure of ARLs (mutation sites ≥ 3), we found that ARL13B had more mutation sites than others (Figure 2B). Moreover, we discovered varying degrees of genetic variation among the 22 ARLs (1.7% to 10.0%), and the mutation ratios of ARL4A, ARL13B and ARL16 were relatively higher, up to 10.0% (Figure S2A). We further checked the alteration frequency of ARLs (mutation ratio ≥ 8%) in various GC types. As shown in Figure 2C, copy‐number amplification obviously contributed to the mRNA expression alteration of ARLs in different GC types. More interestingly, DNA methylation analysis demonstrated that there was a negative correlation between mRNA expression and DNA methylation for most ARLs (R ≥ 0.3, *P* < 0.05) (Figure 2D). A recent study showed that deregulation of ARL4C was due to hypomethylation in its 3’‐UTR in lung squamous cell carcinoma.^27^ Therefore, we further investigated the specific methylation sites of ARL4C using MEXPRESS tool and find that DNA methylation status of cg24441922 and cg11509907 sites of the 3’‐UTR was significantly negatively related to ARL4C mRNA expression in GC (Figure S2B and Table S4). Taken together, these results suggested that DNA methylation was also involved in the epigenetic regulation of ARLs in GC.

### 3.3 The diagnostic and prognostic values of ARLs for GC

**FIGURE 3:**
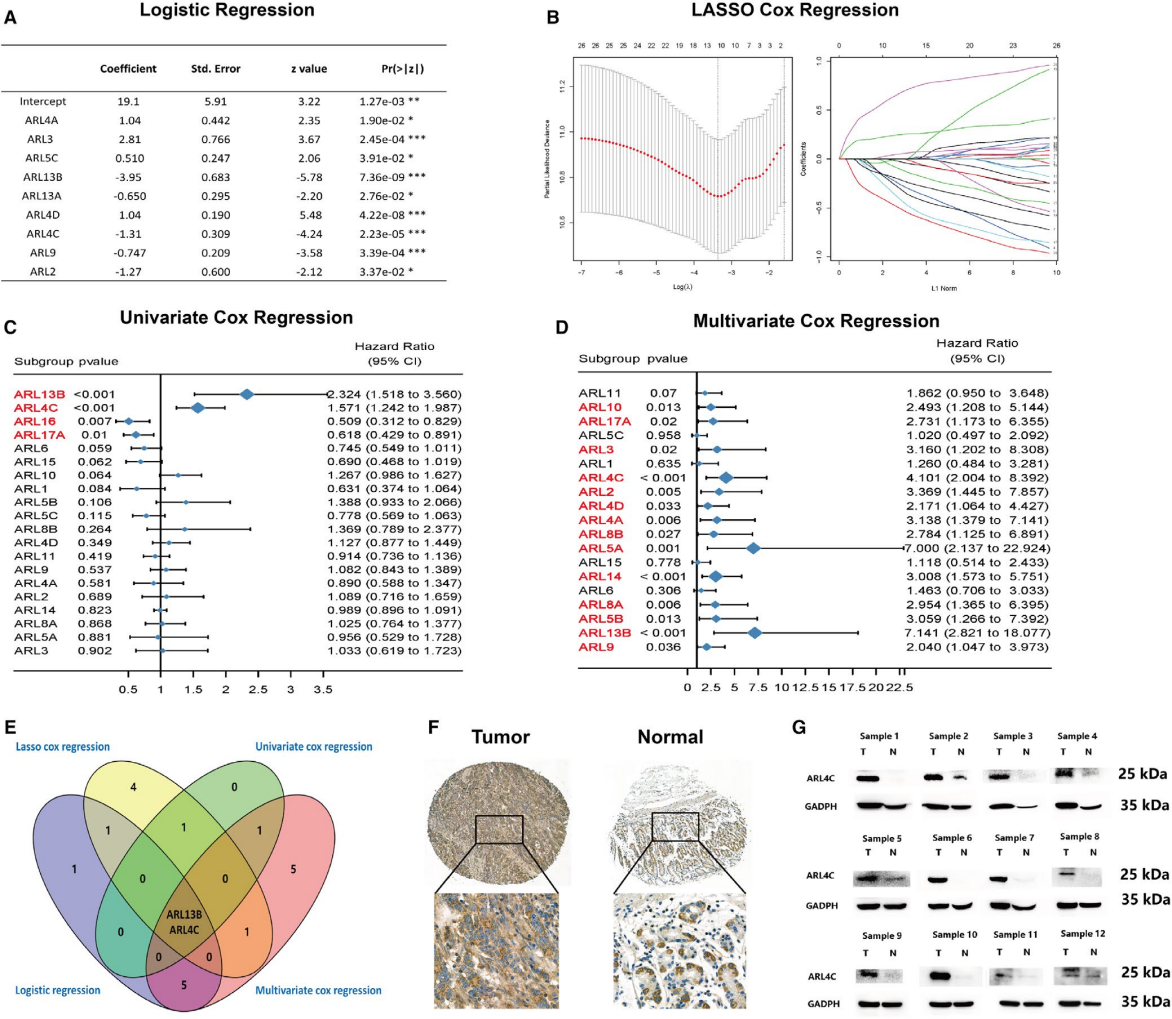
Analysis of the diagnostic and prognostic values of ARLs in GC. A, Logistic regression analysis of ARLs in GC. B, LASSO coefficient profiles of the OS‐related ARLs. A coefficient profile plot was produced against the log λ sequence. Vertical line was drawn at the value selected using 10‐fold cross‐validation, where optimal lambda results in 22 nonzero coefficients. C, The forest plot showed the distribution of OS hazard ratios across ARLs in GC patients in univariate Cox model. D, The forest plot showed the distribution of OS hazard ratios across ARLs in GC patients in multivariate Cox model. E, The Venn diagram showed that four machine learning models jointly identified ARL13B and ARL4C as the two most critical diagnostic and prognostic indicators for GC. F, Representative images of IHC staining of ARL4C in GC and adjacent normal tissues. Scale bars represent 500 μm (low magnification) and 100 μm (high magnification). G, Representative images of Western blot analysis of ARL4C expression in frozen tumour and adjacent mucosa tissues of 12 GC patients from Xijing Hospital of Digestive Diseases. Each experiment was carried out at least three times

We further assessed the diagnostic and prognostic values of ARLs in GC patients based on the TCGA, GTEx and GEO data sets. Firstly, we constructed the logistic regression model to test the usefulness of ARLs in GC diagnosis. All samples were randomly administrated into training (75%) and validation (25%) cohorts. All ARLs in the training cohort were identified and featured with nonzero coefficients by logistic regression model. Then, diagnostic markers with high significance were selected using the stepwise method ('both' method). As shown in Figure 3A, we identified nine ARLs as the potential diagnostic markers for GC. Moreover, we evaluated the ability of predicted diagnostic markers in differentiating the GC patients from the normal in validation cohort. The result suggested that our selected diagnostic markers had a high accuracy of prediction (area under the curve (AUC) = 0.929) (Figure S3A).

Furthermore, we analysed the effects of ARLs on the overall survival (OS) of GC patients using the Kaplan‐Meier (K‐M) plotter. We observed that 9 ARLs (*ie* ARL1, ARL4C, ARL5A, ARL5B, ARL9, ARL13B, ARL15, ARL17A and ARL17B) were significantly related to patient prognosis (Figure S3B). Thus, ARLs were of great significance for assessing prognosis for GC patients. Then, we further identified the key prognostic markers for the purpose of avoiding overfitting of the predictive model with the minimum criteria via constructing LASSO Cox regression model (Figure 3B and Figure S3C), where eight ARLs (ARL1, ARL4C, ARL5C, ARL6, ARL13B, ARL14, ARL15 and ARL16) were selected that were reliably associated with OS. The univariate and multivariate Cox regression models were also undertaken to study the prognostic values of all ARLs for GC (Figure 3C,D). After integrating diagnostic analysis and prognostic analysis results, we acknowledged that ARL4C and ARL13B were the two most important diagnostic and prognostic markers for GC patients among all ARLs (Figure 3E).

ARL13B has been previously reported to play a critical role in promoting proliferation, migration and invasion of GC cells and is associated with poor prognosis of GC patients.^17^ However, the biological functions of ARL4C in gastric tumorigenesis remained unclear. Therefore, we evaluated the protein expression of ARL4C by IHC in a cohort of 142 GC patients (Cohort Ⅰ). Higher ARL4C expression was found in primary GC samples compared with normal gastric mucosa tissues (Figure 3F). Furthermore, we identified the ARL4C expression in frozen tumour and adjacent mucosa tissues of 12 GC patients at the Xijing Hospital of Digestive Diseases (Cohort Ⅱ) by Western blot analysis. The results indicated that the protein expression of ARL4C in the tumour tissues was significantly higher than that in the adjacent mucosa tissues (Figure 3G). Meanwhile, ARL4C overexpression could remarkably dampen the prognosis of GC patients after adjusting for several confounding factors, including subtype, Lauren classification, stage, age at surgery and gender (Figure S3D).

### 3.4 ARL4C knockdown inhibited the proliferation, invasion, metastasis and EMT (epithelial‐mesenchymal transition) in GC

**FIGURE 4:**
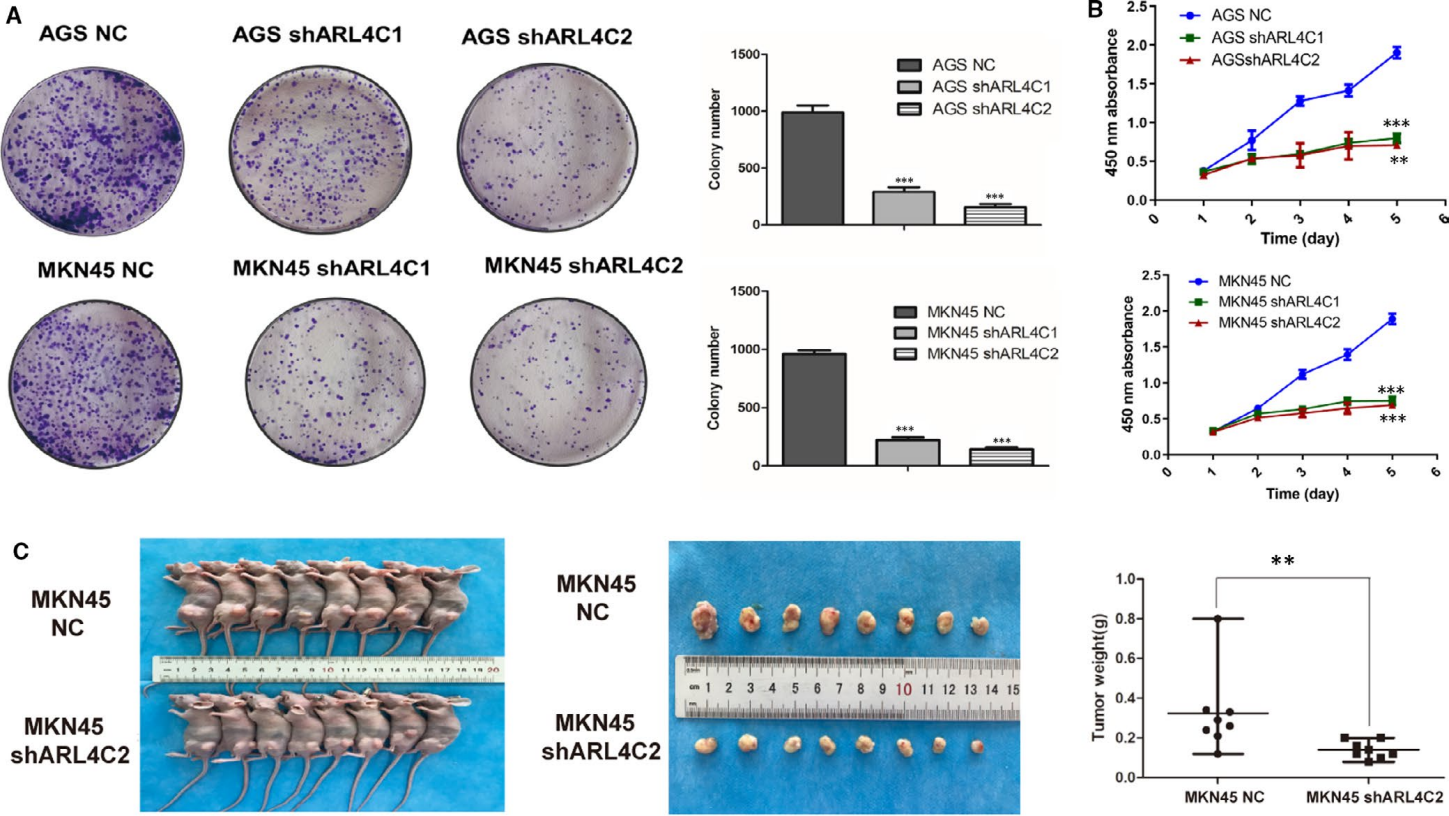
Silencing of ARL4C inhibited the proliferation capacity of GC cells. A, Both shARL4C #1 and shARL4C #2 transduction could remarkably reduce the size and number of clones formed by AGS and MKN45 cells. B, Both shARL4C #1 and shARL4C #2 transduction inhibited the cell viability of AGS and MKN45 cells. C, Representative images of tumours in nude mice (n = 8) implanted with MKN45 cells expressing shARL4C #2 or negative control (NC). The weights of the xenograft tumours were measured. **P < 0.01, ***P < 0.001

**FIGURE 5:**
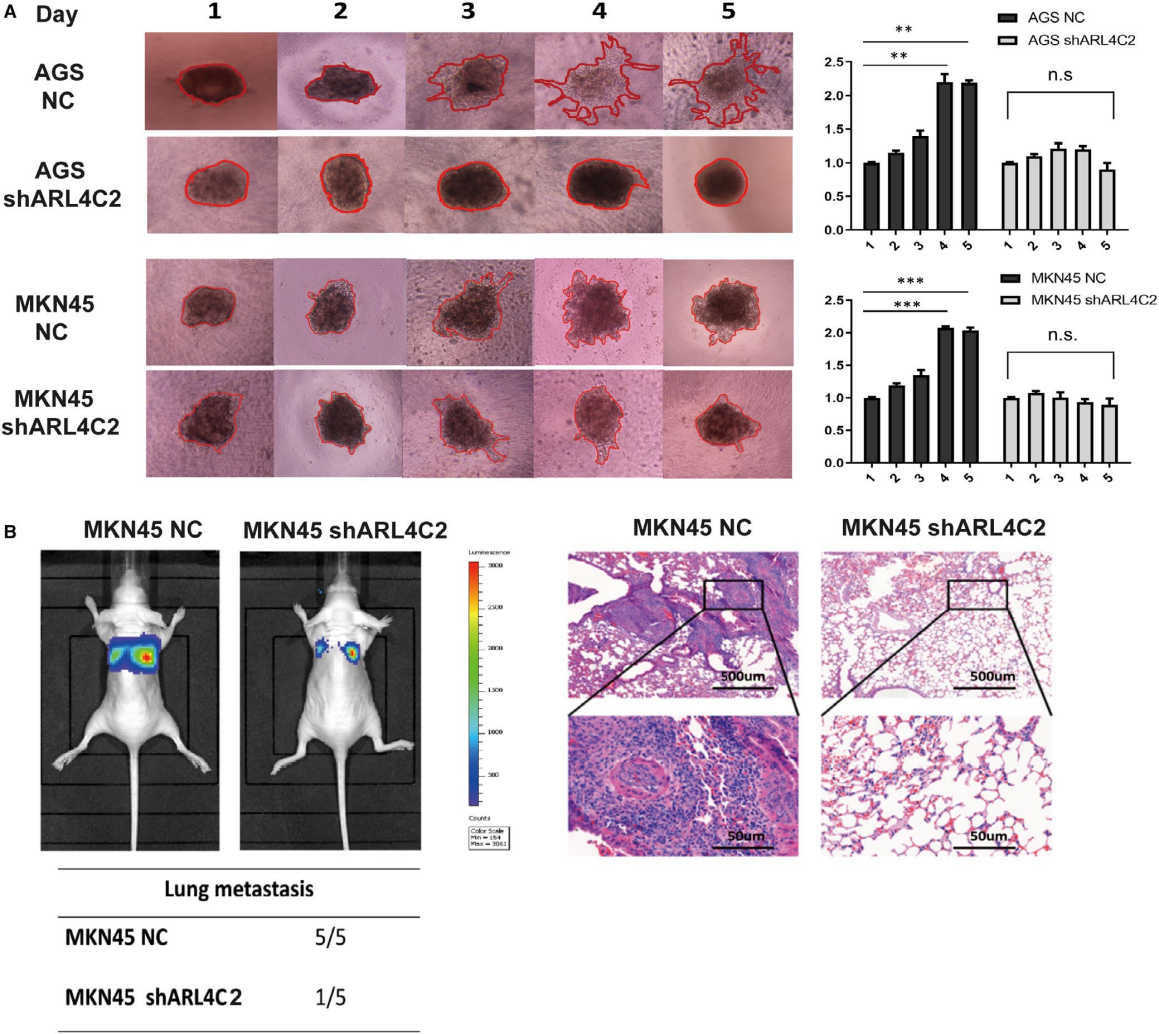
Silencing of ARL4C inhibited the invasive capacity of GC cells in 3D culture and in vivo. A, Down‐regulating ARL4C inhibited the invasive ability of AGS and MKN45 cells in 3D culture. Photographs of all the spheroids in each well every 24 h for 5 d used a 4 × objective. Quantitative analysis of the surface area of all spheroids. Normalized areas for all the spheroids were presented relative to the area on the first day. B, Representative bioluminescent imaging (BLI) of the different groups following tail vein injection (10 wk). Representative H&E staining of lung tissues from the different groups. The incidences of lung metastases in the different groups of nude mice. Each experiment was carried out at least three times. **P < 0.01, ***P < 0.001

Given that ARL4C was involved in regulating the biological behaviours of various tumours, we examined whether its expression might affect the malignant phenotypes of GC cells by in vitro and in vivo experiments. In contrast to other small G proteins, ARL4C activity is putatively regulated by its expression level rather than the switch between GDP‐ and GTP‐bound status induced by regulators.^13^ Thus, we explored the role of ARL4C in the tumorigenesis of GC by constructing ARL4C knockdown GC cells. AGS and MKN45 cells were transduced with 2 shRNAs (shARL4C #1 and shARL4C #2) against ARL4C, and the knockdown efficacy was confirmed by RT‐PCR and Western blot analyses. As shown in Figure S4A, shARL4C #2 was chosen to perform 3D invasion assay and in vivo experiments for its higher knockdown efficacy. CCK‐8 assays revealed that ARL4C down‐regulation significantly reduced cell growth compared with the negative control (NC) (Figure 4A), which was further confirmed by colony forming assays (Figure 4B). Furthermore, the in vivo analysis showed that silencing ARL4C in MKN45 cells caused obvious reductions in tumour weight and volume in nude mice (Figure 4C). The 3D invasion experiment, as shown in Figure 5A, indicated that ARL4C knockdown can decrease the invasion ability of GC cells in 3D culture. The in vivo metastatic assay also indicated that the down‐regulation of ARL4C decreased the incidence of lung metastasis and the size of metastatic lung nodules (Figure 5B). Overall, these results suggested that ARL4C might play a critical role in GC growth and metastasis in vitro and in vivo.

**FIGURE 6:**
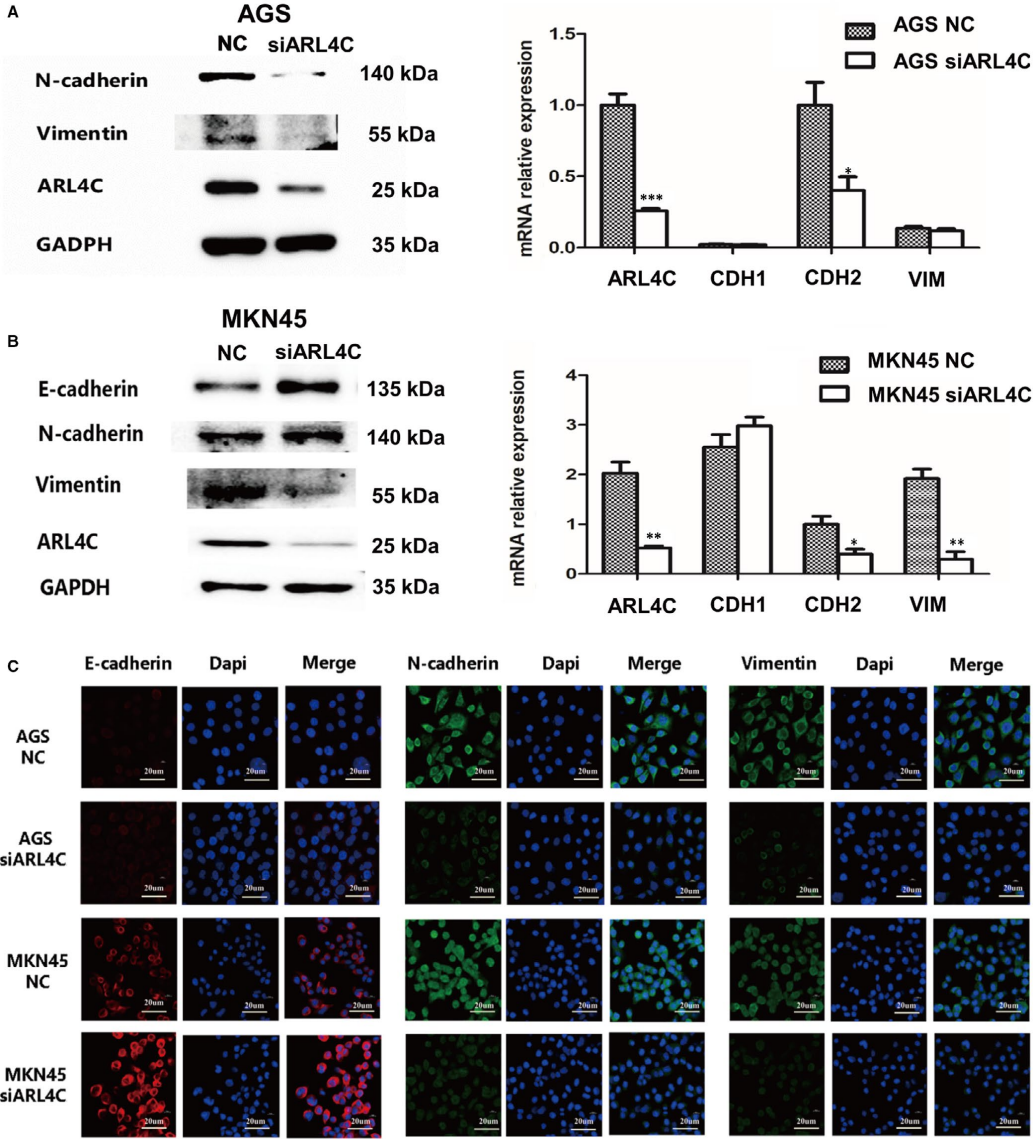
Silencing of ARL4C inhibited EMT of GC cells. A, Representative images of Western blot and RT‐PCR showed the changes in mesenchymal markers (CDH2/N‐cadherin and VIM/vimentin) after ARL4C silencing in AGS cells. B, Representative images of Western blot and RT‐PCR showed the changes in epithelial marker (CDH1/ E‐cadherin) and mesenchymal markers (CDH2/N‐cadherin and VIM/vimentin) after ARL4C silencing in MKN45 cells. C, After transfected with siARL4C or NC, AGS and MKN45 cells were fluorescence‐stained for E‐cadherin (red), N‐cadherin (green), vimentin (green) and DAPI (blue). Each experiment was carried out at least three times

Epithelial‐mesenchymal transition (EMT) is involved in tumour aggressive progression. To confirm the role of ARL4C in regulating EMT of GC cells, we evaluated the expression changes in EMT markers after ARL4C silencing. AGS and MKN45 cells were transfected with 2 siRNAs (siARL4C #1 and siARL4C #2) against ARL4C, and the knockdown efficacy was confirmed by RT‐PCR and Western blot analyses (Figure S4B). We chose siARL4C #2 to perform further experiments for its higher knockdown efficacy. Western blot and RT‐PCR analyses showed that down‐regulation of ARL4C led to the increased expression of E‐cadherin and decreased expression of N‐cadherin and Vimentin compared with the NC group (Figure 6A,B and Figure S5A). Furthermore, the IF assays showed the similar results (Figure 6C). Consistently, in TCGA cohort, ARL4C mRNA expression exhibited a positive correlation with mRNA expression of EMT markers, such as Slug, Snail and Vimentin (Figure S6B). Therefore, ARL4C participated in maintaining the EMT phenotype of GC cells.

### 3.5 ARL4C acted as a mediator of TGF‐β1/Smad signalling in GC

**FIGURE 7:**
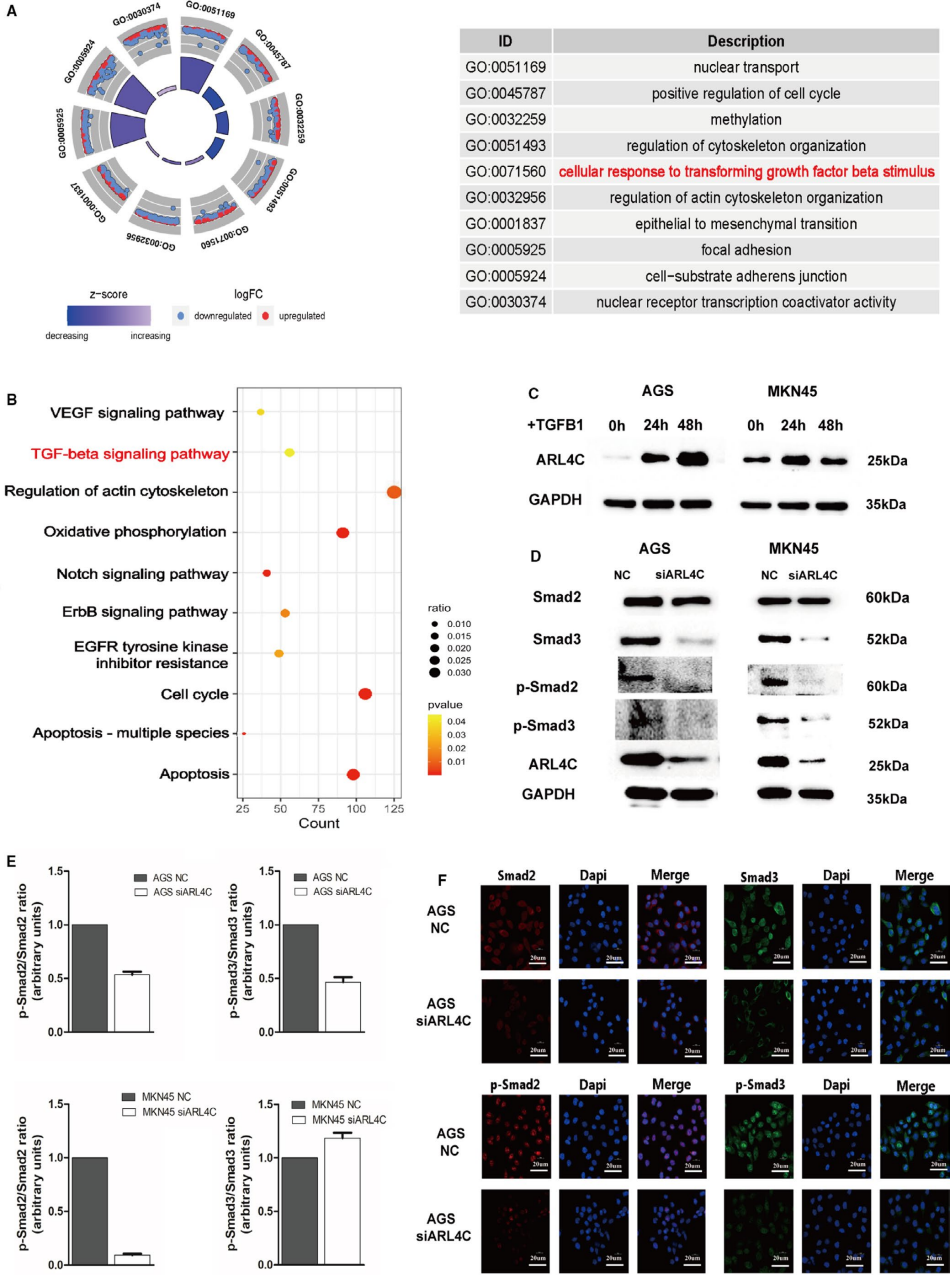
Identification of ARL4C as a mediator for TGF‐β1/Smad signal pathway. A, Circus plot showed the GO enrichment terms of ARL4C‐associated genes in GC. B, KEGG pathway analysis identified the significant association between ARL4C and TGF‐β signalling pathway. C, ARL4C protein expression was enhanced by TGF‐β1 treatment (10 ng/ml) in AGS and MKN45 cells. D, Western blot analysis of expression levels of Smads in AGS and MKN45 cells after silencing ARL4C. E, Relative protein levels in (D) were quantified by ImageJ (presented as the ratio of phospho‐Smad2 to total Smad2 levels and the ratio of phospho‐Smad3 to total Smad3 levels). F, Immunofluorescence analysis of expression levels of Smads in AGS cells after silencing ARL4C. Each experiment was carried out at least three times

To uncover the underlying mechanisms of ARL4C in GC, we explored the TCGA database to identify the genes related to ARL4C. As shown in Figure S6A,C, we identified that numbers of GC‐related genes were highly correlated with ARL4C, among which TGF‐β1 was the most significant gene (R = 0.851, *P* < 0.01). GO and KEGG enrichment analyses indicated that the ARL4C‐associated genes (R ≥ 0.5, *P* < 0.05) were significantly involved in the cellular response to TGF‐β stimulus and TGF‐β signalling pathway (Figure 7A,B).

As TGF‐β1 is identified as an important inducer of the malignant progression of cancer,^28^ we investigated whether ARL4C might participate in TGF‐β1–induced progression of GC. We treated AGS and MKN45 cells with 10 ng/mL TGF‐β1 for 24 and 48 hours. Following TGF‐β1 stimulation, compared with the control, ARL4C was significantly up‐regulated in AGS and MKN45 cells. In particular, TGF‐β1–induced ARL4C expression was in a time‐dependent manner in AGS cells (Figure 7C). In addition, Western blot analysis and IF analysis showed the down‐regulation of ARL4C decreased phosphorylated Smad2 (p‐Smad2) and phosphorylated Smad3 (p‐Smad3) in AGS. In MKN45 cells, Smad2 phosphorylation was significantly inhibited after ARL4C silencing (Figure 7D‐F,S5B,D). Meanwhile, TCGA correlation analysis showed that ARL4C was positively related to Smad2 and Smad3 with high correlation coefficients (Figure S6B). These data suggested that ARL4C might mediate the TGF‐β1/Smad signalling pathway.

### 3.6 ARL4C enhanced the TGF‐β1–mediated poor prognosis of GC patients

**FIGURE 8:**
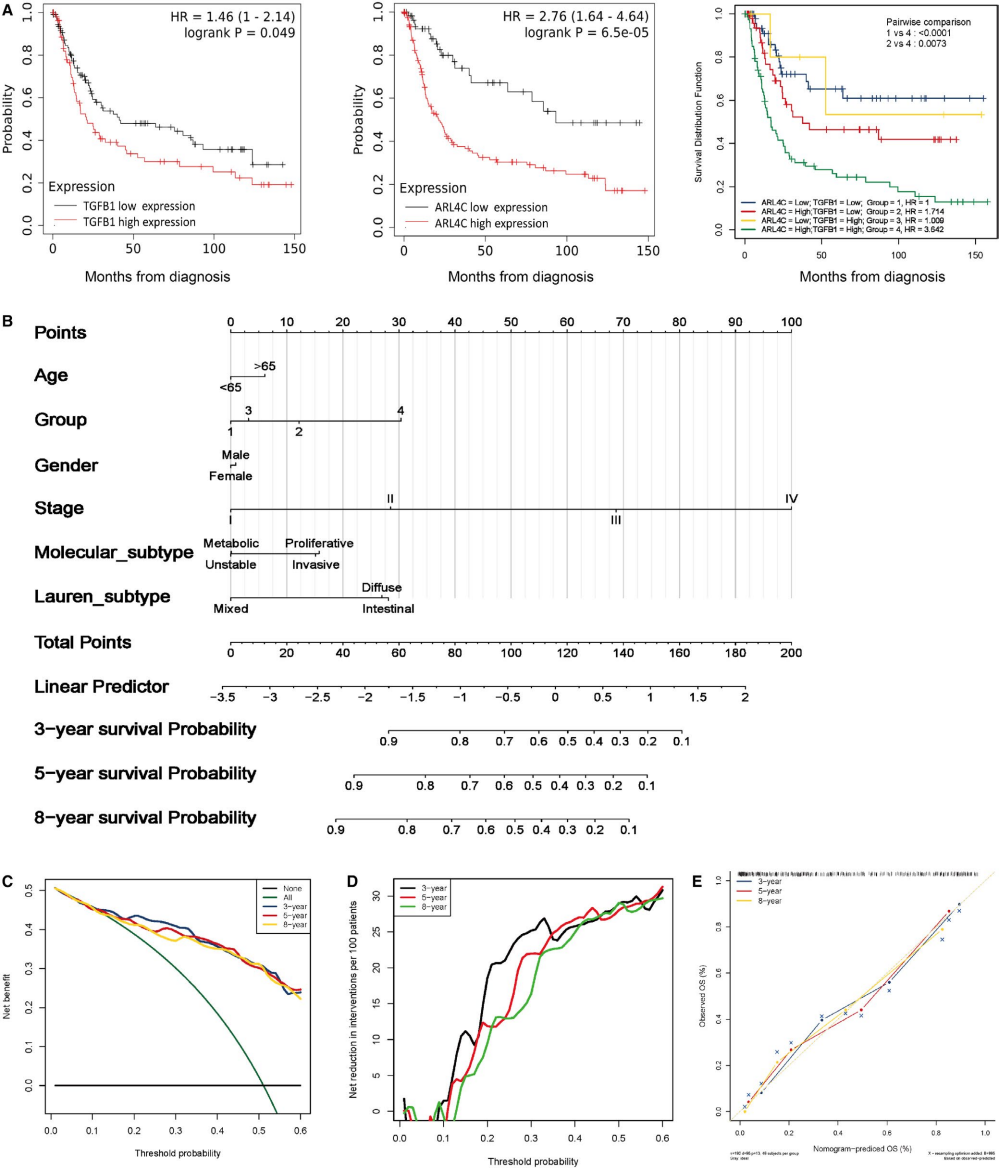
ARL4C enhanced TGF‐β1–mediated poor prognosis in GC. A, Kaplan‐Meier analysis of GC patient survival stratified according to TGF‐β1 and ARL4C in GSE15459 cohort. B, The OM nomogram based on multivariate analysis at 3, 5 or 8 y in GSE15459 cohort. C and D, Decision curve analysis (DCA) for validating the predictive performance of the nomogram at 3, 5 or 8 y. E, The calibration curves for the probability of survival showed an optimal agreement of the prediction by the nomogram at 3, 5 or 8 y

To translate the above findings into clinical significance, we analysed clinical data of ARL4C and TGF‐β1 expression in GC patients from GSE15459 cohort. We divided the samples into 4 groups according to the expression status of ARL4C and TGF‐β1: group 1 (ARL4C^low^/ TGF‐β1^low^), group 2 (ARL4C^high^/ TGF‐β1^low^), group 3 (ARL4C^low^/ TGF‐β1^high^) and group 4 (ARL4C^high^/ TGF‐β1^high^). The Kaplan‐Meier analysis showed that elevated expression of TGF‐β1 or ARL4C was associated with shorter OS of GC patients. Furthermore, patients with coexpression of TGF‐β1 and ARL4C had the lowest OS (Figure 8A).

Next, we constructed a predictive nomogram based on overall mortality (OM) via multivariate Cox regression model. The nomogram incorporated six variables: age, the expression status of TGF‐β1 and ARL4C, gender, stage, molecular subtype and Lauren subtype. As shown in Figure 8B, to evaluate the individual's probability of overall mortality, values for the prognostic factors had to be determined. Each independent prognostic factor was assigned an exact score scale, the points must be added up to obtain the total risk score at 3, 5 and 8 years. The OM probability can be read from the x‐axis (total risk score) to predict the corresponding probabilities of independent prognostic factors on the left y‐axis.

The nomogram demonstrated that stage of GC patients contributed significantly to the individual's probability of OM and patients in stage Ⅳ had the highest mortality. Secondly, the expression status of TGF‐β1 and ARL4C was a critical prognostic factor for GC patients. Group 4 (ARL4C^high^/ TGF‐β1^high^) had the higher probability of OM than group 1 (ARL4C^low^/ TGF‐β1^low^), group 2 (ARL4C^high^/ TGF‐β1^low^) and group 3 (ARL4C^low^/ TGF‐β1^high^) at 3, 5 and 8 years. We then adopted DCA to verify the prognostic accuracy of the nomogram in OM prediction. The results showed that the best net benefit was similar with the prediction of the nomogram at 3, 5 and 8 years (Figure 8C,D).

The calibration curves of the nomogram at 3, 5 and 8 years were very close to the best prediction curve, showing a great consistency between the predicted OM rates and the actual observations (Figure 8E). Taken together, these results suggested that ARL4C was critical for TGF‐β1–mediated poor clinical outcomes for GC patients.

## 4 DISCUSSION

ARLs have been reported to play important roles in cancer progression. Nevertheless, the exact role of ARLs in GC and the underlying molecular mechanisms are not well illustrated. The present study aims to comprehensively understand the expression patterns, correlation, genetic alteration, diagnostic values, prognostic values and potential functions of ARLs in GC by integrated bioinformatics analysis and biological experiments.

The expression profiles of ARLs are firstly explored using TCGA, GTEx and Oncomine databases, which demonstrate that ARLs are commonly dysregulated in GC. Accumulating evidence suggests that the crosstalk and collaboration between small GTPase proteins are involved in several cellular processes and diseases.^25^, ^29^, ^30^, ^31^ In this study, coexpression and correlation analyses demonstrate that the expression levels of ARLs in GC show high correlations. Meanwhile, there are majorities of shared protein domains among ARLs, implying they might possess similar functions. Furthermore, according to the Hallmark gene sets analysis, we discover that ARLs may modulate numbers of cancer‐related pathways, and more interestingly, several ARLs are involved in the same pathways. For instance, ARL5C, ARL10, ARL13B and ARL13A are enriched in p53 pathway, which is critical to GC progression.^32^ Taken together, our bioinformatics analysis indicates that dysregulated ARLs might function synergistically to modulate various signalling pathways in GC.

Our further genetic analysis indicates that the genetic alterations, including copy‐number amplification and DNA methylation status, are involved in the misregulation of ARLs in GC. Particularly, we find that DNA methylation status is inversely correlated with the mRNA expression of several ARLs in GC. It is generally accepted that DNA methylation is a major epigenetic process that plays a critical role in different stages of cancer evolution and development.^33^ Fuji's study shows that the hypomethylation in the 3’‐UTR induces the overexpression of ARL4C in lung cancer, which contributes to the malignant phenotypes of cancer cells.^27^ In line with this, we find that the methylation status at cg24441922 site is significantly negatively related to ARL4C mRNA expression in GC. Overall, we acknowledge that DNA methylation status might be involved in the dysregulation and oncogenic functions of ARLs in GC.

Multiple machine learning models are constructed to evaluate the diagnostic and prognostic values of ARLs in GC. After comprehensively analysing the logistic regression model, univariate Cox regression model, multivariate Cox regression model and LASSO Cox regression model, we firstly reveal that ARL4C and ARL13B are the two most critical indicators for diagnosis and prognosis in GC among all ARLs. Consistent with our results, recent studies have uncovered that overexpression of ARL13B and ARL4C is correlated strongly with the poor prognosis of GC patients.^14^, ^17^ ARL13B may worsen the survival and stimulate GC cell proliferation and migration both in vitro and in vivo. Meanwhile, it might regulate Smo trafficking and activate the Hedgehog signalling pathway.^17^ On the other hand, in vitro assays suggest that ARL4C knockdown would inhibit the migration capacity of GC cells under 2D culture and reduce protein expression of Slug, which is related to EMT.^14^ However, a previous study has shown that overexpression of ARL4C could promote cancer cell proliferation in 3D and in vivo experiments, while the 2D assays could not yield the similar results in colon cancer and lung cancer.^13^ Therefore, we perform both in vitro and in vivo experiments to further assess the effects of ARL4C on the growth and metastasis of GC cells. Our results support a close association between ARL4C expression and GC malignant phenotypes. Down‐regulation of ARL4C could inhibit cell proliferation and metastasis both in vitro and in vivo, and ARL4C knockdown could inhibit EMT phenotype, as indicated by the increased expression of epithelial marker (E‐cadherin) and decreased expression of mesenchymal markers (N‐cadherin and vimentin).

TGF‐β1, as a pleiotropic cytokine, orchestrates complicated signals to modulate tumorigenesis and promote cancer progression.^34^ Increasing preclinical and clinical studies have identified TGF‐β signalling as a determinant in immunotherapy.^35^ TCGA data mining in our study indicates TGF‐β1 as the most significant ARL4C‐related gene in GC. Further functional enrichment analysis also demonstrates that the ARL4C‐associated genes in GC are significantly linked to the TGF‐β–related signalling. Thus, we speculate that ARL4C could participate in TGF‐β1 pathway. Accordingly, the expression of ARL4C is significantly up‐regulated with the stimulation of TGF‐β1 in GC cells, while knockdown of ARL4C weakens Smad2 phosphorylation level. Interestingly, we find that ARL4C silencing could decrease both totally expression level and phosphorylation level of Smad3. It has been reported that down‐regulation of ARL4C might prevent nuclear localization of YAP/TAZ in lung cancer cells and colorectal cancer cells.^13^ Meanwhile, knockdown of YAP/TAZ could impair phosphorylation level and Smad3 transcriptional activity of Smad3.^36^ Accordingly, we speculate that knockdown of ARL4C could dampen both total expression level and phosphorylation level of Smad3 by inhibiting nuclear localization of YAP/TAZ in GC cells. These results indicate that ARL4C may act as a mediator between TGF‐β1 and Smads in GC. Remarkably, the Kaplan‐Meier analysis shows that ARL4C/TGF‐β1 coexpression is associated with shorter OS of GC patients. To perform prognostic prediction more precisely, we further construct an OM nomogram based on the expression status of ARL4C and TGF‐β1 and other clinical variables. Consistently, our nomogram indicates that the ARL4C/TGF‐β1 coexpression is an independent risk factor and associates with the highest mortality at 3, 5 and 8 years. In sum, ARL4C may act as a mediator of TGF‐β1/Smad signalling and enhance the TGF‐β1‐mediated poor prognosis in GC. Our results demonstrate the great promise of ARL4C targeting treatment in improving the effectiveness of TGF‐β1 inhibitors for GC patients.

## 5 CONCLUSION

To our knowledge, it is for the first time that the expression patterns, genetic alterations, signalling pathways and clinical values of ARLs in GC have been fully explored. Our results identify ARL4C as one of the two most significant diagnostic and prognostic indicators in GC. ARL4C down‐regulation might suppress the proliferation, metastasis and EMT of GC cells. Furthermore, ARL4C could function as a mediator of TGF‐β1 signalling and enhance TGF‐β1‐associated poor prognosis in GC. Our studies provide an overall insight into the specific roles of ARLs that benefits the development of novel strategies for GC detection and treatment.

## CONFLICT OF INTEREST

The authors declare no conflict of interests.

## AUTHOR CONTRIBUTIONS

Ning Xie: Experimental work and data analysis; co‐authoring of the manuscript. Yuru Bai: Immunohistochemistry and cell experiments. Jian Wu: Authoring of the manuscript. Yan Li: CCK‐8 and data analysis. Mingzuo Jiang: 3D‐invasion assay and RT‐PCR analyses. Bing Xu: 3D‐invasion assay and RT‐PCR analyses. Zhen Ni: Some animal experiments. Ting Yuan: Some animal experiments. Feng Xu: Authoring of the manuscript. Yongquan Shi: Authoring of the manuscript. Kaichun Wu: Funding. Jinhai Wang: Funding. Lei Dong: Funding; project coordination; authoring of the manuscript. Na Liu: Funding; authoring of the manuscript. Yunfan Bai and Lu Qiao contributed equally to this work. All authors reviewed the results and approved the final version of the manuscript.

## Supporting information

Figure S1‐S6 and Table S1‐S8

Supporting information

## Data Availability

The data that support the findings of this study are available from the corresponding author upon reasonable request.
