# Supplementary material for: ARL4C might serve as a prognostic factor and a novel therapeutic target for gastric cancer: bioinformatics analyses and biological experiments
Source: J Cell Mol Med. 2021 Mar 16;25(8):4014–27. doi: 10.1111/jcmm.16366 (PMC8051716; doi:10.1111/jcmm.16366)
Supplement: Supplementary file 1 — Figure S1‐S6 and Table S1‐S8 [file JCMM-25-4014-s001.pdf]

Supplementary Information

Figure S1.

The expression profiles of ARLs in Oncomine database.

Figure S2.

The OncoPrint visual summarised of genetic variations of ARLs.

DNA methylation status of ARL4C in GC.

Figure S3.

OS analysis of ARLs for GC.

The AUC of Logistic Regression model.

Lasso Cox regression normalization results.

Multivariate Cox regression analysis of ARL4C in GC.

Figure S4.

Knockdown efficacy of siARL4Cs and Lv-shARL4Cs in GC cells.

Figure S5.

siARL4C#1 and siARL4C#2 transfection could affect the expression of EMT makers and Smads.

Figure S6.

Correlation analysis of ARL4C-related genes in TCGA dataset.

Table S1: Correlation coefficients among ARLs in GC.

Table S2: The enrichment results of ARLs in GSVA hallmark pathways.

Table S3: Chromosome locations of ARLs (GRCh38/hg38).

Table S4: DNA methylation status of ARL4C in GC.

Table S5: Primer sequences used for RT-PCR.

Table S6: siRNA sequences for ARL4C.

Table S7: Clinical samples information of GC tissue microarray

Table S8: Clinical samples information from Xijing Hospital of Digestive Diseases

Supplementary Figure 1

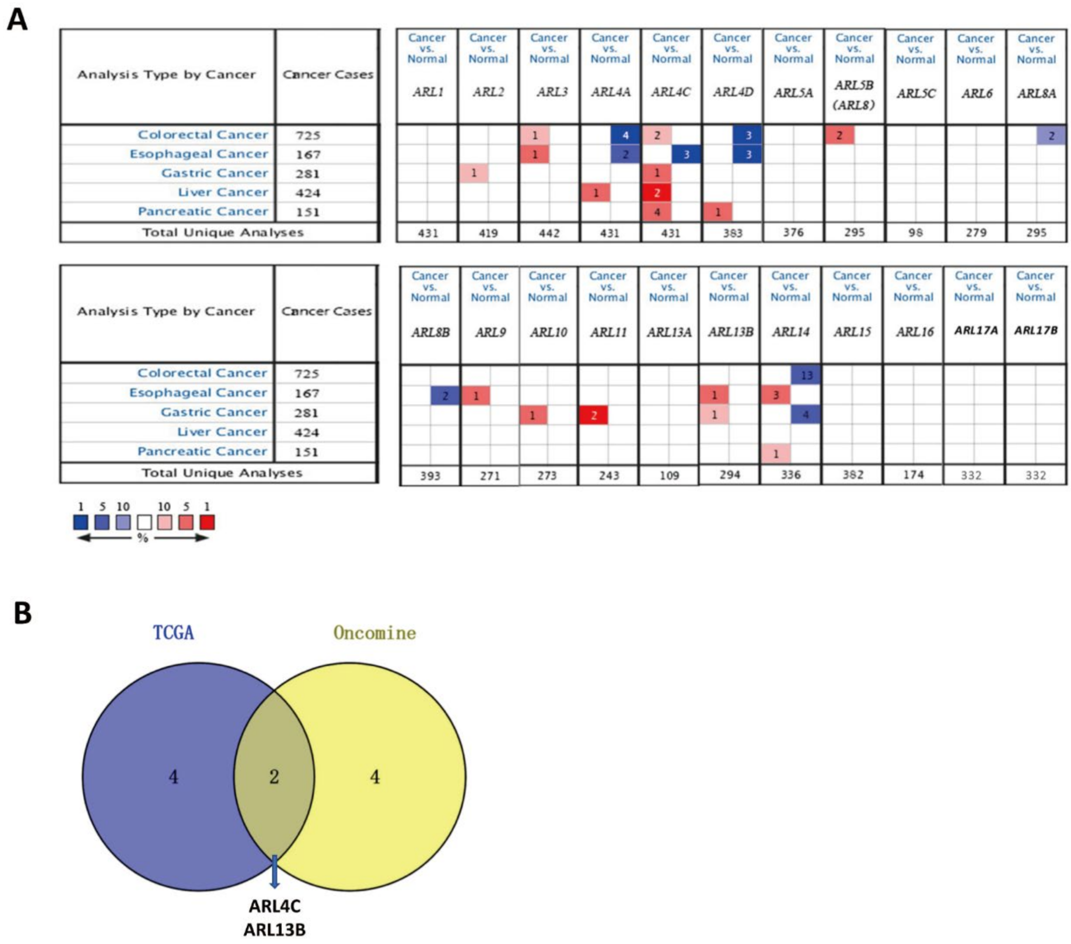

Figure S1. (A) The expression profiles of ARLs in digestive system cancer using Oncomine dataset. **(B)** ARL4C and ARL13B were dysregulated in GC in both TCGA and Oncomine datasets.

Supplementary Figure 2

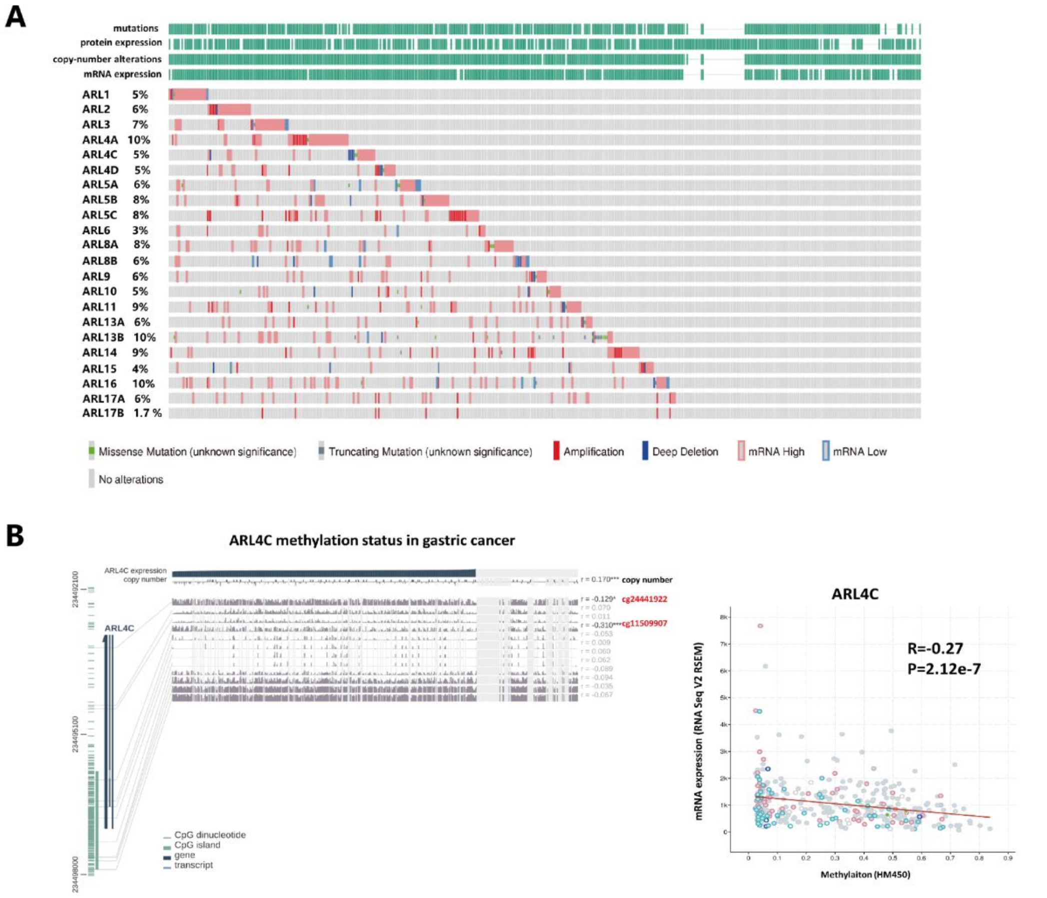

Figure S2. (A) The OncoPrint visual summarised of genetic variations of ARLs in TCGA database. **(B)** DNA methylation status of ARL4C in GC (*P < 0.05; **P < 0.01).

Supplementary Figure 3

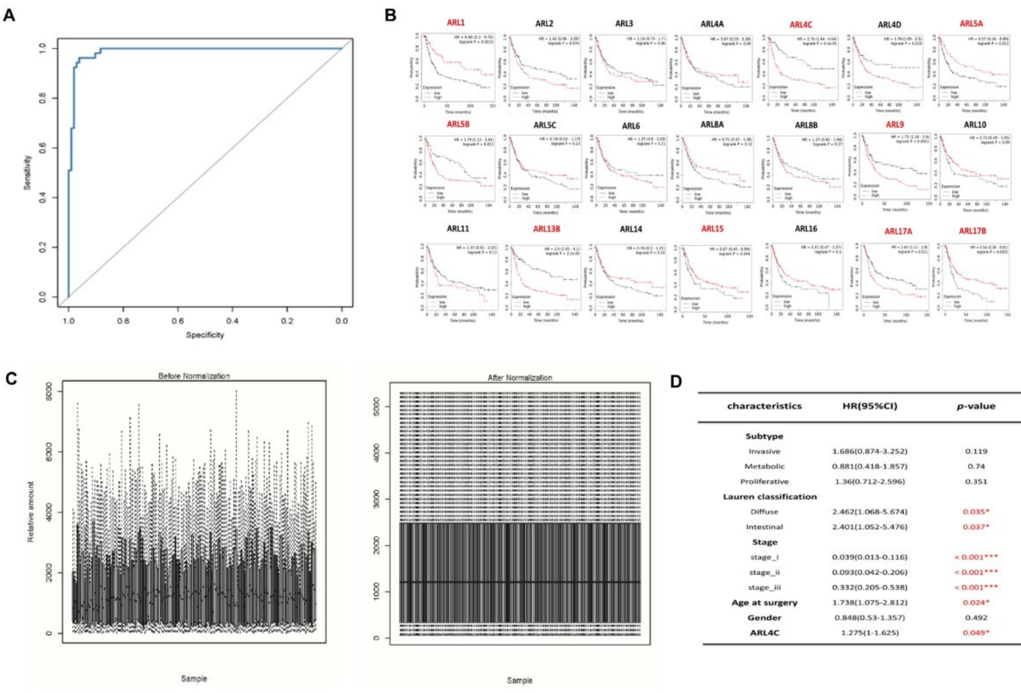

characteristics	HR(95%CI)	*p*-value
**Subtype**		
Invasive	1.686(0.874-3.252)	0.119
Metabolic	0.881(0.418-1.857)	0.74
Proliferative	1.36(0.712-2.596)	0.351
**Lauren classification**		
Diffuse	2.462(1.068-5.674)	0.035*
Intestinal	2.401(1.052-5.476)	0.037*
**Stage**		
stage_i	0.039(0.013-0.116)	< 0.001***
stage_ii	0.093(0.042-0.206)	< 0.001***
stage_iii	0.332(0.205-0.538)	< 0.001***
**Age at surgery**	1.738(1.075-2.812)	0.024*
**Gender**	0.848(0.53-1.357)	0.492
**ARL4C**	1.275(1-1.625)	0.049*

Figure S3. (A) The AUC evaluated the accuracy of Logistic Regression model in validation cohort. **(B)** OS analysis of ARLs for GC using Kaplan–Meier plotter. **(C)** Lasso Cox regression normalization results. **(D)** Multivariate Cox regression analysis of ARL4C in GC (*P＜0.05, **P＜0.01, ***P＜0.001).

Supplementary Figure 4

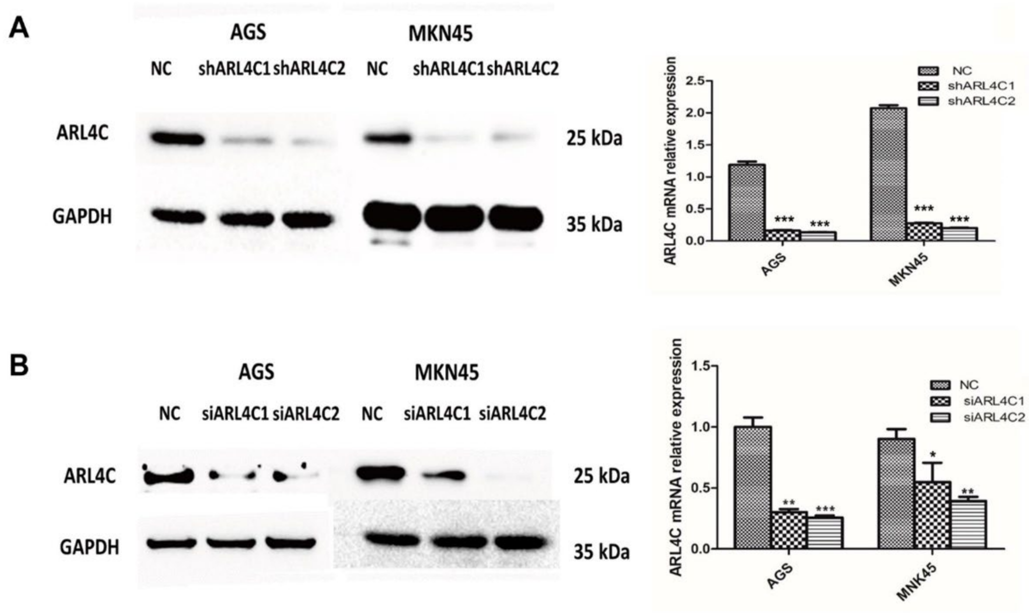

Figure S4. **(A)** Western blot and RT-PCR showed mRNA (right) and protein (left) expression levels of ARL4C in AGS and MKN45 cells transduced with LV-shARL4C (shARL4C #1 and shARL4C #2) and NC. ShARL4C #2 had better knockdown efficacy than shARL4C #1 in both AGS and MKN45 cells. **(B)** Western blot and RT-PCR showed the mRNA (right) and protein (left) expression levels of ARL4C in AGS and MKN45 cells transfected with siARL4Cs (siARL4C #1 and siARL4C #2) and negative control (NC). The results indicated that siARL4C #2 had better knockdown efficacy than siARL4C #1. *P<0.05, **P<0.01, ***P<0.001.

Supplementary Figure 5

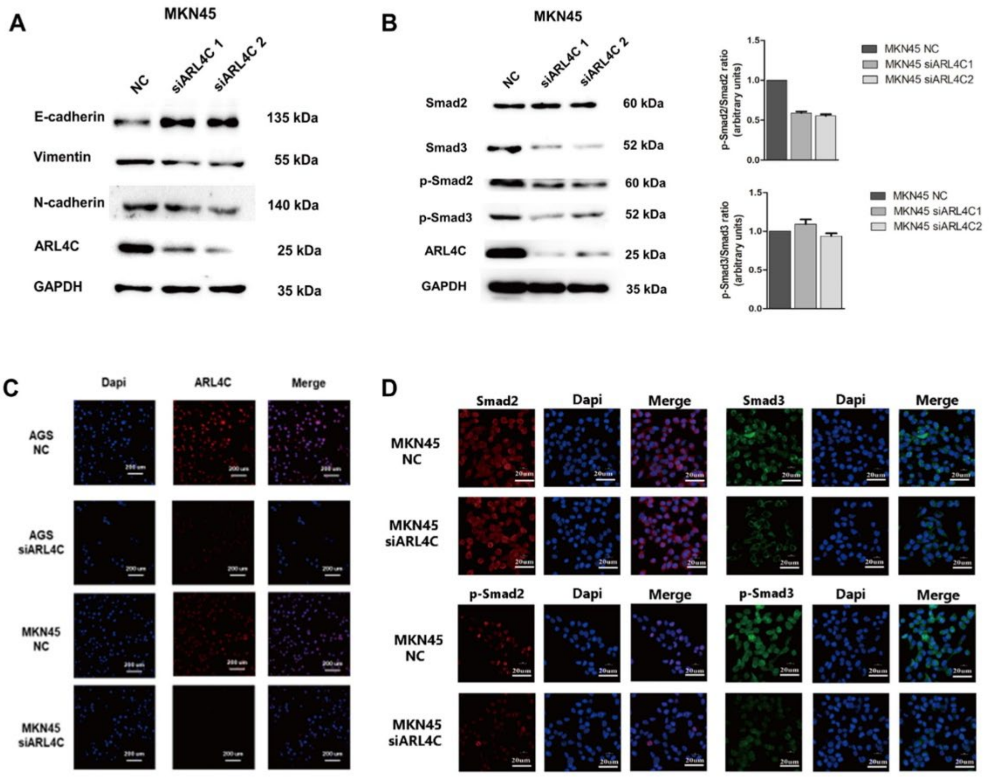

Figure S5. (A) Western blot assays showed changes of epithelial marker (E-cadherin) and mesenchymal markers (N-cadherin and Vimentin) after ARL4C silencing by siARL4C #1 and siARL4C #2 in MKN45 cells. **(B)** Western blot analysis of expression levels of Smads in MKN45 cells after ARL4C silencing by siARL4C #1 and siARL4C #2. **(C)** Immunofluorescence analysis of expression levels of ARL4C in AGS and MKN45 cells after ARL4C silencing. **(D)** Immunofluorescence analysis of expression levels of Smads in MKN45 cells after ARL4C silencing.

Supplementary Figure 6

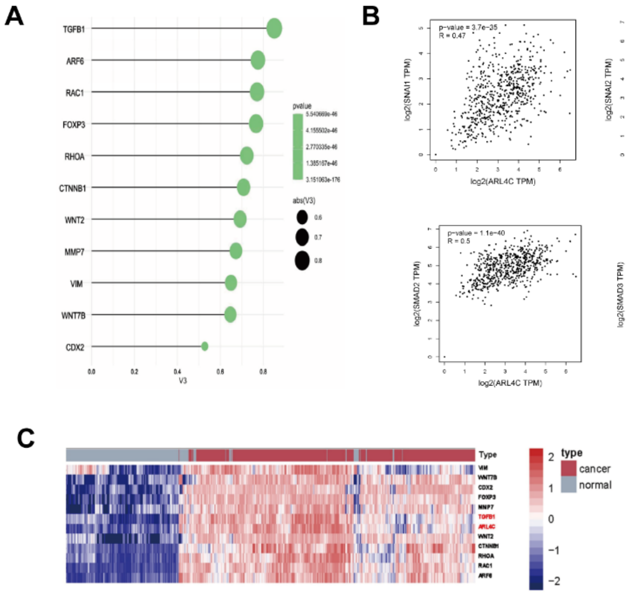

Figure S6. Enrichment analysis of TCGA database. (A) The Correlation bar showed the Pearson's correlation coefficients of ARL4C-related genes in TCGA dataset. **(B)** The scatter plots showed the correlation coefficients between ARL4C with SNAIL1, SNAIL2, VIM, SMAD2 and SMAD3, respectively. **(C)** The heat map showed the differential expression of genes mentioned in **(A)**.

Table S1: Correlation coefficients among ARLs in GC.

a	b	coefficient
ARL6	ARL1	0.233925
ARL6	ARL4A	0.135747
ARL6	ARL8B	0.37178
ARL6	ARL3	0.363742
ARL6	ARL5C	0.052109
ARL6	ARL8A	-0.09999
ARL6	ARL11	0.068277
ARL6	ARL5A	0.246023
ARL6	ARL5B	0.143493
ARL6	ARL13B	0.506276
ARL6	ARL13A	0.178775
ARL6	ARL10	0.344937
ARL6	ARL4D	0.161859
ARL6	ARL14	-0.02989
ARL6	ARL15	0.260374
ARL6	ARL17A	0.144953
ARL6	ARL4C	0.155692
ARL6	ARL9	-0.01601
ARL6	ARL2	-0.0716
ARL6	ARL16	0.099088
ARL6	ARL17B	0.071294
ARL1	ARL4A	0.003373
ARL1	ARL8B	0.331241
ARL1	ARL3	0.073313
ARL1	ARL5C	-0.05443
ARL1	ARL8A	-0.14831
ARL1	ARL11	-0.11943
ARL1	ARL5A	0.304047
ARL1	ARL5B	0.257989
ARL1	ARL13B	0.037293
ARL1	ARL13A	-0.02536
ARL1	ARL10	-0.11946
ARL1	ARL4D	-0.16124
ARL1	ARL14	0.112517
ARL1	ARL15	0.204034

ARL1	ARL17A	0.005966
ARL1	ARL4C	-0.11091
ARL1	ARL9	0.026669
ARL1	ARL2	-0.28304
ARL1	ARL16	-0.00227
ARL1	ARL17B	-0.07847
ARL4A	ARL8B	0.191724
ARL4A	ARL3	0.039554
ARL4A	ARL5C	0.101671
ARL4A	ARL8A	0.047878
ARL4A	ARL11	-0.05562
ARL4A	ARL5A	0.142368
ARL4A	ARL5B	0.300399
ARL4A	ARL13B	0.186507
ARL4A	ARL13A	0.152399
ARL4A	ARL10	-0.0789
ARL4A	ARL4D	-0.03948
ARL4A	ARL14	0.06962
ARL4A	ARL15	0.042024
ARL4A	ARL17A	0.014632
ARL4A	ARL4C	0.099182
ARL4A	ARL9	-0.05794
ARL4A	ARL2	-0.11888
ARL4A	ARL16	0.098103
ARL4A	ARL17B	-0.02397
ARL8B	ARL3	0.05096
ARL8B	ARL5C	-0.03009
ARL8B	ARL8A	-0.02809
ARL8B	ARL11	0.059399
ARL8B	ARL5A	0.313218
ARL8B	ARL5B	0.252668
ARL8B	ARL13B	0.286926
ARL8B	ARL13A	0.038624
ARL8B	ARL10	0.246915
ARL8B	ARL4D	0.230334
ARL8B	ARL14	-0.00437
ARL8B	ARL15	0.165981
ARL8B	ARL17A	0.149443
ARL8B	ARL4C	0.14868
ARL8B	ARL9	-0.07792
ARL8B	ARL2	-0.19319
ARL8B	ARL16	-0.04192
ARL8B	ARL17B	0.074685
ARL3	ARL5C	-0.04152

ARL3	ARL8A	0.110299
ARL3	ARL11	0.068364
ARL3	ARL5A	0.021285
ARL3	ARL5B	-0.12107
ARL3	ARL13B	0.233194
ARL3	ARL13A	-0.03735
ARL3	ARL10	0.197011
ARL3	ARL4D	0.204258
ARL3	ARL14	-0.17512
ARL3	ARL15	0.087636
ARL3	ARL17A	0.004036
ARL3	ARL4C	0.106887
ARL3	ARL9	0.022365
ARL3	ARL2	0.284892
ARL3	ARL16	0.202535
ARL3	ARL17B	0.133291
ARL5C	ARL8A	-0.086
ARL5C	ARL11	0.05408
ARL5C	ARL5A	0.046765
ARL5C	ARL5B	0.025674
ARL5C	ARL13B	0.157149
ARL5C	ARL13A	0.436491
ARL5C	ARL10	0.09173
ARL5C	ARL4D	0.003539
ARL5C	ARL14	0.048151
ARL5C	ARL15	-0.05165
ARL5C	ARL17A	0.165266
ARL5C	ARL4C	-0.05083
ARL5C	ARL9	0.048055
ARL5C	ARL2	-0.04264
ARL5C	ARL16	0.121354
ARL5C	ARL17B	0.254366
ARL8A	ARL11	0.075128
ARL8A	ARL5A	-0.21633
ARL8A	ARL5B	-0.05918
ARL8A	ARL13B	0.044527
ARL8A	ARL13A	-0.14598
ARL8A	ARL10	-0.12611
ARL8A	ARL4D	-0.02168
ARL8A	ARL14	-0.06892
ARL8A	ARL15	-0.11009
ARL8A	ARL17A	-0.1641
ARL8A	ARL4C	0.215142
ARL8A	ARL9	-0.03337

ARL8A	ARL2	0.295827
ARL8A	ARL16	0.041978
ARL8A	ARL17B	-0.13014
ARL11	ARL5A	0.015903
ARL11	ARL5B	0.047956
ARL11	ARL13B	0.199836
ARL11	ARL13A	0.172208
ARL11	ARL10	0.036099
ARL11	ARL4D	-0.09434
ARL11	ARL14	-0.11651
ARL11	ARL15	0.02105
ARL11	ARL17A	0.127801
ARL11	ARL4C	0.058235
ARL11	ARL9	-0.06976
ARL11	ARL2	0.044883
ARL11	ARL16	0.049355
ARL11	ARL17B	0.075373
ARL5A	ARL5B	0.37417
ARL5A	ARL13B	0.133657
ARL5A	ARL13A	0.106726
ARL5A	ARL10	0.064037
ARL5A	ARL4D	-0.03686
ARL5A	ARL14	-0.02174
ARL5A	ARL15	0.244179
ARL5A	ARL17A	0.202495
ARL5A	ARL4C	0.075436
ARL5A	ARL9	0.052764
ARL5A	ARL2	-0.17671
ARL5A	ARL16	0.011421
ARL5A	ARL17B	0.116231
ARL5B	ARL13B	0.214569
ARL5B	ARL13A	0.095128
ARL5B	ARL10	0.002492
ARL5B	ARL4D	-0.14362
ARL5B	ARL14	-0.03021
ARL5B	ARL15	0.131883
ARL5B	ARL17A	0.123866
ARL5B	ARL4C	0.093727
ARL5B	ARL9	0.064305
ARL5B	ARL2	-0.18705
ARL5B	ARL16	-0.07697
ARL5B	ARL17B	-0.07976
ARL13B	ARL13A	0.310408
ARL13B	ARL10	0.282557

ARL13B	ARL4D	0.113314
ARL13B	ARL14	0.013721
ARL13B	ARL15	0.055987
ARL13B	ARL17A	0.221271
ARL13B	ARL4C	0.090017
ARL13B	ARL9	0.020024
ARL13B	ARL2	-0.06696
ARL13B	ARL16	0.179839
ARL13B	ARL17B	0.268378
ARL13A	ARL10	0.249908
ARL13A	ARL4D	-0.00603
ARL13A	ARL14	-0.06977
ARL13A	ARL15	0.065102
ARL13A	ARL17A	0.486676
ARL13A	ARL4C	-0.07022
ARL13A	ARL9	-0.00082
ARL13A	ARL2	-0.11386
ARL13A	ARL16	0.155312
ARL13A	ARL17B	0.43133
ARL10	ARL4D	0.408917
ARL10	ARL14	-0.20448
ARL10	ARL15	0.153902
ARL10	ARL17A	0.352367
ARL10	ARL4C	0.109538
ARL10	ARL9	-0.02128
ARL10	ARL2	0.008394
ARL10	ARL16	-0.04308
ARL10	ARL17B	0.23374
ARL4D	ARL14	-0.12174
ARL4D	ARL15	-0.12827
ARL4D	ARL17A	0.092054
ARL4D	ARL4C	0.056735
ARL4D	ARL9	-0.07726
ARL4D	ARL2	0.126094
ARL4D	ARL16	-0.06468
ARL4D	ARL17B	0.123137
ARL14	ARL15	-0.0191
ARL14	ARL17A	-0.00146
ARL14	ARL4C	-0.16206
ARL14	ARL9	-0.0311
ARL14	ARL2	-0.19259
ARL14	ARL16	0.026623
ARL14	ARL17B	-0.04396
ARL15	ARL17A	-0.03995

ARL15	ARL4C	0.127786
ARL15	ARL9	0.010922
ARL15	ARL2	-0.11641
ARL15	ARL16	-0.02233
ARL15	ARL17B	-0.07843
ARL17A	ARL4C	-0.02735
ARL17A	ARL9	-0.08446
ARL17A	ARL2	-0.06347
ARL17A	ARL16	0.170028
ARL17A	ARL17B	0.544132
ARL4C	ARL9	0.031856
ARL4C	ARL2	0.176758
ARL4C	ARL16	-0.03213
ARL4C	ARL17B	-0.13002
ARL9	ARL2	0.011734
ARL9	ARL16	0.004204
ARL9	ARL17B	-0.03231
ARL2	ARL16	0.134995
ARL2	ARL17B	-0.0031
ARL16	ARL17B	0.099158

Table S2: The enrichment results of ARLs in GSVA hallmark pathways.

Hallmark pathways	col	V3
CHOLESTEROL_HOMEOSTASIS	ARL6	-0.31192
GLYCOLYSIS	ARL6	-0.30493
OXIDATIVE_PHOSPHORYLATION	ARL6	-0.30026
REACTIVE_OXIGEN_SPECIES_PATHWAY	ARL6	-0.34418
UV_RESPONSE_DN	ARL6	0.399817
XENOBIOTIC_METABOLISM	ARL6	-0.34297
ANDROGEN_RESPONSE	ARL1	0.453818
APICAL_JUNCTION	ARL1	-0.33418
APICAL_SURFACE	ARL1	-0.34401
MYOGENESIS	ARL1	-0.36359
PROTEIN_SECRETION	ARL1	0.569163
PROTEIN_SECRETION	ARL8B	0.384238
HEDGEHOG_SIGNALING	ARL3	0.311139
ADIPOGENESIS	ARL5C	-0.34862
ESTROGEN_RESPONSE_EARLY	ARL5C	-0.3635
ESTROGEN_RESPONSE_LATE	ARL5C	-0.35115
HEME_METABOLISM	ARL5C	-0.30341
P53_PATHWAY	ARL5C	-0.32881
PI3K_AKT_MTOR_SIGNALING	ARL5C	-0.32774
UV_RESPONSE_UP	ARL8A	0.383563
WNT_BETA_CATENIN_SIGNALING	ARL8A	0.305802
CHOLESTEROL_HOMEOSTASIS	ARL5A	-0.34469
COAGULATION	ARL5A	-0.30552
ESTROGEN_RESPONSE_LATE	ARL5A	-0.37645
G2M_CHECKPOINT	ARL5A	0.304407
ANGIOGENESIS	ARL5B	-0.40688
APICAL_JUNCTION	ARL5B	-0.31855
E2F_TARGETS	ARL5B	0.422976
G2M_CHECKPOINT	ARL5B	0.513261
KRAS_SIGNALING_DN	ARL5B	-0.33472
MITOTIC_SPINDLE	ARL5B	0.413187
MYOGENESIS	ARL5B	-0.48065
PROTEIN_SECRETION	ARL5B	0.305288
SPERMATOGENESIS	ARL5B	0.363525
UNFOLDED_PROTEIN_RESPONSE	ARL5B	0.302812
CHOLESTEROL_HOMEOSTASIS	ARL13B	-0.36183
P53_PATHWAY	ARL13B	-0.30785
XENOBIOTIC_METABOLISM	ARL13B	-0.4052
ADIPOGENESIS	ARL13A	-0.40078
APICAL_JUNCTION	ARL13A	-0.33383
CHOLESTEROL_HOMEOSTASIS	ARL13A	-0.47957

COAGULATION	ARL13A	-0.34487
ESTROGEN_RESPONSE_LATE	ARL13A	-0.34502
FATTY_ACID_METABOLISM	ARL13A	-0.37962
GLYCOLYSIS	ARL13A	-0.3368
OXIDATIVE_PHOSPHORYLATION	ARL13A	-0.38667
P53_PATHWAY	ARL13A	-0.39518
REACTIVE_OXIGEN_SPECIES_PATHWAY	ARL13A	-0.35985
UV_RESPONSE_UP	ARL13A	-0.38655
XENOBIOTIC_METABOLISM	ARL13A	-0.40441
APICAL_SURFACE	ARL10	0.320402
CHOLESTEROL_HOMEOSTASIS	ARL10	-0.33879
DNA_REPAIR	ARL10	-0.54752
E2F_TARGETS	ARL10	-0.40366
EPITHELIAL_MESENCHYMAL_TRANSITION	ARL10	0.397333
FATTY_ACID_METABOLISM	ARL10	-0.47339
GLYCOLYSIS	ARL10	-0.50619
HEDGEHOG_SIGNALING	ARL10	0.440575
HYPOXIA	ARL10	0.402906
KRAS_SIGNALING_DN	ARL10	0.356673
MTORC1_SIGNALING	ARL10	-0.54897
MYC_TARGETS_V1	ARL10	-0.50188
MYC_TARGETS_V2	ARL10	-0.40014
MYOGENESIS	ARL10	0.378952
OXIDATIVE_PHOSPHORYLATION	ARL10	-0.59731
P53_PATHWAY	ARL10	-0.41846
PEROXISOME	ARL10	-0.42309
REACTIVE_OXIGEN_SPECIES_PATHWAY	ARL10	-0.46008
UNFOLDED_PROTEIN_RESPONSE	ARL10	-0.43864
UV_RESPONSE_DN	ARL10	0.565397
UV_RESPONSE_UP	ARL10	-0.31113
XENOBIOTIC_METABOLISM	ARL10	-0.47061
KRAS_SIGNALING_DN	ARL4D	0.384636
MYOGENESIS	ARL4D	0.377611
COMPLEMENT	ARL15	0.364091
IL2_STAT5_SIGNALING	ARL15	0.388111
IL6_JAK_STAT3_SIGNALING	ARL15	0.342674
INFLAMMATORY_RESPONSE	ARL15	0.302571
KRAS_SIGNALING_UP	ARL15	0.456184
NOTCH_SIGNALING	ARL15	0.330982
TGF_BETA_SIGNALING	ARL15	0.341549
UV_RESPONSE_DN	ARL15	0.423956
ADIPOGENESIS	ARL17A	-0.32019
CHOLESTEROL_HOMEOSTASIS	ARL17A	-0.32682
OXIDATIVE_PHOSPHORYLATION	ARL17A	-0.3538

UV_RESPONSE_UP	ARL17A	-0.31092
APOPTOSIS	ARL4C	0.34372
EPITHELIAL_MESENCHYMAL_TRANSITION	ARL4C	0.406189
IL2_STAT5_SIGNALING	ARL4C	0.315191
INFLAMMATORY_RESPONSE	ARL4C	0.330085
INTERFERON_GAMMA_RESPONSE	ARL4C	0.342447
NOTCH_SIGNALING	ARL4C	0.347079
TGF_BETA_SIGNALING	ARL4C	0.376194
TNFA_SIGNALING_VIA_NFKB	ARL4C	0.317884
ANDROGEN_RESPONSE	ARL2	-0.38073
MYOGENESIS	ARL2	0.313822
PROTEIN_SECRETION	ARL2	-0.40186
ESTROGEN_RESPONSE_EARLY	ARL16	-0.3395
ADIPOGENESIS	ARL17B	-0.32289
CHOLESTEROL_HOMEOSTASIS	ARL17B	-0.32072
ESTROGEN_RESPONSE_EARLY	ARL17B	-0.30222
GLYCOLYSIS	ARL17B	-0.37379
UNFOLDED_PROTEIN_RESPONSE	ARL17B	-0.31639
UV_RESPONSE_UP	ARL17B	-0.34754

Table S3: Chromosome locations of ARLs (GRCh38/hg38).

	**Gene position GRCh38/hg38**	**Size**	**Orientation**
ARL1	chr12:101,393,116-101,407,820	14,705 bases	Minus strand
ARL2	chr11:65,014,113-65,022,185	8,073 bases	Plus strand
ARL3	chr10:102,673,727-102,714,433	40,707 bases	Minus strand
ARL4A	chr7:12,686,827-12,690,958	4,132 bases	Plus strand
ARL4C	chr2:234,493,041-234,497,081	4,041 bases	Minus strand
ARL4D	chr17:43,398,985-43,401,137	2,153 bases	Plus strand
ARL5A	chr2:151,788,984-151,828,495	39,512 bases	Minus strand
ARL5B	chr10:18,659,335-18,681,639	22,305 bases	Plus strand
ARL5C	chr17:39,156,894-39,167,484	10,591 bases	Minus strand
ARL6	chr3:97,764,521-97,801,242	36,722 bases	Plus strand
ARL8A	chr1:202,133,404-202,144,743	11,340 bases	Minus strand
ARL8B	chr3:5,122,245-5,180,916	58,672 bases	Plus strand
ARL9	chr4:56,505,209-56,525,481	20,273 bases	Plus strand
ARL10	chr5:176,365,474-176,415,463	49,990 bases	Plus strand
ARL11	chr13:49,628,299-49,633,872	5,574 bases	Plus strand
ARL13A	chrX:100,969,040-100,990,831	21,792 bases	Plus strand
ARL13B	chr3:93,980,134-94,055,678	75,545 bases	Plus strand
ARL14	chr3:160,677,160-160,678,448	1,289 bases	Plus strand
ARL15	chr5:53,883,942-54,310,586	426,645 bases	Minus strand
ARL16	chr17:81,681,155-81,683,924	2,770 bases	Minus strand
ARL17A	chr17:46,499,818-46,579,792	79,975 bases	Minus strand
ARL17B	chr17:46,274,184-46,362,064	87,881 bases	Minus strand

Table S4: DNA methylation status of ARL4C in GC.

variable	p-value	Pearson_r
cnv	0.000957	0.170116498
cg24441922	0.017581	-0.1290858
cg05204104	0.198602	0.070098165
cg15016771	0.842517	0.010846352
cg11509907	7.02E-09	-0.310072923
cg21650900	0.329907	-0.053154522
cg21460828	0.862269	0.009471307
cg04942334	0.270089	0.060156164
cg13539030	0.257327	0.0617826
cg09453076	0.102406	-0.088993488
cg05308656	0.084736	-0.0939057
cg15235893	0.52393	-0.034782856
cg09935994	0.216105	-0.067454671

Table S5: Primer sequences used for RT-PCR.

mRNA	Forward	Reverse
ARL4C	GCAGTAAAGTAAAGCCCTGTGGTG	GGTCAGAGACGAAACGGGCTA
CDH1	AGGATGACACCCGGGACAAC	TGCAGCTGGCTCAAGTCAAAG
CDH2	AGCACAGTGGCCACCTACAAAG	CAGCTCCTGGCCCAGTTACA
VIM	AACCTGGCCGAGGACATCA	TCAAGGTCAAGACGTGCCAGA

Note: Sequences are listed 5'- 3'.

Table S6: **siRNA sequences for ARL4C.**

siRNAs	Sense	Antisense
siARL4C#1	GUAGGUCAUUAUCACACAATT	UUGUGUGAUAAUGACCUACTT
siARL4C#2	GCAGGAUAGUUAAAGGUGUTT	ACACCUUUAACUAUCCUGCTT

Note: Sequences are listed 5'- 3'.

Table S7: Clinical samples information of GC tissue microarray

No.	Age	Sex	Organ	Pathology diagnosis	Grade	TNM Stage	Stage
A1	47	F	Stomach	Adenocarcinoma	1	T2N0M0	Ib
A2	66	M	Stomach	Mucinous adenocarcinoma	3	T3N0M0	II
A3	70	M	Stomach	Adenocarcinoma	2	T2N0M0	Ib
A4	66	M	Stomach	Adenocarcinoma	2	T3N0M0	II
A5	62	M	Stomach	Adenocarcinoma	2	T3N0M0	II
A6	49	M	Stomach	Adenocarcinoma	2	T3N0M0	II
A7	57	M	Stomach	Adenocarcinoma	2	T2N0M0	Ib
A8	66	M	Stomach	Adenocarcinoma	2	T1N0M0	Ia
A9	43	M	Stomach	Adenocarcinoma	3	T2N0M0	Ib
A10	54	F	Stomach	Adenocarcinoma	2	T2N0M0	Ib
A11	44	M	Stomach	Adenocarcinoma	2	T2N0M0	Ib
A12	57	M	Stomach	Adenocarcinoma	3	T2N0M0	Ib
A13	58	M	Stomach	Adenocarcinoma	2	T2N0M0	Ib
A14	64	M	Stomach	Adenocarcinoma	3	T2N0M0	Ib
A15	50	F	Stomach	Adenocarcinoma	3	T3N0M0	II
B1	47	F	Stomach	Adenocarcinoma	1	T2N0M0	Ib
B2	66	M	Stomach	Mucinous adenocarcinoma	3	T3N0M0	II
B3	70	M	Stomach	Adenocarcinoma	2	T2N0M0	Ib
B4	66	M	Stomach	Adenocarcinoma	2	T3N0M0	II
B5	62	M	Stomach	Adenocarcinoma	2	T3N0M0	II
B6	49	M	Stomach	Adenocarcinoma	2	T3N0M0	II
B7	57	M	Stomach	Adenocarcinoma	2	T2N0M0	Ib
B8	66	M	Stomach	Adenocarcinoma	2	T1N0M0	Ia
B9	43	M	Stomach	Adenocarcinoma	3	T2N0M0	Ib
B10	54	F	Stomach	Adenocarcinoma	2	T2N0M0	Ib
B11	44	M	Stomach	Adenocarcinoma	2	T2N0M0	Ib
B12	57	M	Stomach	Adenocarcinoma	3	T2N0M0	Ib
B13	58	M	Stomach	Adenocarcinoma	2	T2N0M0	Ib
B14	64	M	Stomach	Adenocarcinoma (smooth muscle)	-	T2N0M0	Ib
B15	50	F	Stomach	Adenocarcinoma	3	T3N0M0	II
C1	73	M	Stomach	Adenocarcinoma	2	T2N0M0	Ib
C2	76	M	Stomach	Adenocarcinoma	3	T2N0M0	Ib
C3	94	M	Stomach	Adenocarcinoma	2	T2N0M0	Ib
C4	54	M	Stomach	Adenocarcinoma	3	T3N0M0	II
C5	75	M	Stomach	Adenocarcinoma	3	T2N0M0	Ib
C6	56	M	Stomach	Adenocarcinoma	3	T2N0M0	Ib
C7	64	M	Stomach	Adenocarcinoma	3	T2N1M0	II
C8	48	M	Stomach	Adenocarcinoma	3	T2N0M0	Ib
C9	74	M	Stomach	Adenocarcinoma	3	T4N0M1	IV
C10	45	F	Stomach	Adenocarcinoma	3	T2N1M0	II
C11	58	M	Stomach	Adenocarcinoma	2	T2N1M0	II
C12	70	M	Stomach	Adenocarcinoma	3	T2N0M0	Ib
C13	47	F	Stomach	Adenocarcinoma	2	T2N0M0	Ib
C14	58	M	Stomach	Adenocarcinoma	2	T3N0M0	II
C15	32	M	Stomach	Adenocarcinoma	3	T2N0M0	Ib
D1	73	M	Stomach	Adenocarcinoma	2	T2N0M0	Ib
D2	76	M	Stomach	Adenocarcinoma	3	T2N0M0	Ib
D3	94	M	Stomach	Adenocarcinoma	2	T2N0M0	Ib
D4	54	M	Stomach	Adenocarcinoma	3	T3N0M0	II
D5	75	M	Stomach	Adenocarcinoma	3	T2N0M0	Ib
D6	56	M	Stomach	Adenocarcinoma	3	T2N0M0	Ib
D7	64	M	Stomach	Adenocarcinoma	3	T2N1M0	II
D8	48	M	Stomach	Adenocarcinoma	3	T2N0M0	Ib
D9	74	M	Stomach	Adenocarcinoma	3	T4N0M1	IV
D10	45	F	Stomach	Adenocarcinoma	3	T2N1M0	II
D11	58	M	Stomach	Adenocarcinoma	2	T2N1M0	II
D12	70	M	Stomach	Adenocarcinoma	3	T2N0M0	Ib
D13	47	F	Stomach	Adenocarcinoma	2	T2N0M0	Ib
D14	58	M	Stomach	Adenocarcinoma	2	T3N0M0	II
D15	32	M	Stomach	Adenocarcinoma	3	T2N0M0	Ib
E1	82	M	Stomach	Adenocarcinoma	3	T2N0M0	Ib
E2	61	F	Stomach	Adenocarcinoma	3	T3N0M0	II
E3	65	M	Stomach	Adenocarcinoma	3	T4N0M0	IIIa

E4	40	M	Stomach	Adenocarcinoma	3	T4N0M0	IIIa
E5	69	M	Stomach	Adenocarcinoma	3	T2N0M0	Ib
E6	53	M	Stomach	Adenocarcinoma	3	T2N1M0	II
E7	47	M	Stomach	Adenocarcinoma	3	T2N0M0	Ib
E8	49	M	Stomach	Adenocarcinoma	3	T2N0M0	Ib
E9	66	F	Stomach	Adenocarcinoma	3	T3N0M0	II
E10	60	M	Stomach	Adenocarcinoma	3	T2N0M0	Ib
E11	43	F	Stomach	Adenocarcinoma	3	T3N0M0	II
E12	77	M	Stomach	Adenocarcinoma	3	T3N0M0	II
E13	64	M	Stomach	Adenocarcinoma	3	T3N1M0	IIIa
E14	42	M	Stomach	Adenocarcinoma	3	T2N0M0	Ib
E15	50	M	Stomach	Adenocarcinoma	3	T2N0M0	Ib
F1	82	M	Stomach	Adenocarcinoma	3	T2N0M0	Ib
F2	61	F	Stomach	Adenocarcinoma	3	T3N0M0	II
F3	65	M	Stomach	Adenocarcinoma	3	T4N0M0	IIIa
F4	40	M	Stomach	Adenocarcinoma	3	T4N0M0	IIIa
F5	69	M	Stomach	Adenocarcinoma	3	T2N0M0	Ib
F6	53	M	Stomach	Adenocarcinoma	3	T2N1M0	II
F7	47	M	Stomach	Adenocarcinoma	3	T2N0M0	Ib
F8	49	M	Stomach	Adenocarcinoma	3	T2N0M0	Ib
F9	66	F	Stomach	Adenocarcinoma	3	T3N0M0	II
F10	60	M	Stomach	Adenocarcinoma	3	T2N0M0	Ib
F11	43	F	Stomach	Adenocarcinoma	3	T3N0M0	II
F12	77	M	Stomach	Adenocarcinoma	3	T3N0M0	II
F13	64	M	Stomach	Adenocarcinoma	3	T3N1M0	IIIa
F14	42	M	Stomach	Adenocarcinoma	3	T2N0M0	Ib
F15	50	M	Stomach	Adenocarcinoma	3	T2N0M0	Ib
G1	60	F	Stomach	Adenocarcinoma	3	T2N0M0	Ib
G2	66	F	Stomach	Adenocarcinoma	3	T1N1M0	Ib
G3	40	F	Stomach	Adenocarcinoma (sparse)	3	T2N0M0	Ib
G4	55	M	Stomach	Adenocarcinoma	3	T2N0M0	Ib
G5	58	M	Stomach	Adenocarcinoma	3	T1N0M0	Ia
G6	68	F	Stomach	Adenocarcinoma	3	T2N1M0	II
G7	70	M	Stomach	Adenocarcinoma	3	T2N0M0	Ib
G8	54	M	Stomach	Adenocarcinoma	3	T2N0M0	Ib
G9	58	M	Stomach	Adenocarcinoma	3	T1N0M0	Ia
G10	55	M	Stomach	Adenocarcinoma	3	T3N0M0	II
G11	47	F	Stomach	Adenocarcinoma	3	T3N1M0	IIIa
G12	35	M	Stomach	Adenocarcinoma	3	T3N1M0	IIIa
G13	58	M	Stomach	Adenocarcinoma	3	T1N0M0	Ia
G14	59	M	Stomach	Adenocarcinoma	3	T2N0M0	Ib
G15	69	M	Stomach	Adenocarcinoma	3	T2N0M0	Ib
H1	60	F	Stomach	Adenocarcinoma	3	T2N0M0	Ib
H2	66	F	Stomach	Adenocarcinoma	3	T1N1M0	Ib
H3	40	F	Stomach	Adenocarcinoma	3	T2N0M0	Ib
H4	55	M	Stomach	Adenocarcinoma	3	T2N0M0	Ib
H5	58	M	Stomach	Adenocarcinoma	3	T1N0M0	Ia
H6	68	F	Stomach	Adenocarcinoma	3	T2N1M0	II
H7	70	M	Stomach	Adenocarcinoma	3	T2N0M0	Ib
H8	54	M	Stomach	Adenocarcinoma	3	T2N0M0	Ib
H9	58	M	Stomach	Adenocarcinoma	3	T1N0M0	Ia
H10	55	M	Stomach	Adenocarcinoma	3	T3N0M0	II
H11	47	F	Stomach	Adenocarcinoma	3	T3N1M0	IIIa
H12	35	M	Stomach	Adenocarcinoma	3	T3N1M0	IIIa
H13	58	M	Stomach	Adenocarcinoma	3	T1N0M0	Ia
H14	59	M	Stomach	Adenocarcinoma	3	T2N0M0	Ib
H15	69	M	Stomach	Adenocarcinoma	3	T2N0M0	Ib
I1	66	M	Stomach	Adenocarcinoma (sparse)	2	T2N0M0	Ib
I2	60	M	Stomach	Adenocarcinoma	3	T3N0M0	II
I3	56	M	Stomach	Adenocarcinoma	2	T2N0M0	Ib
I4	56	M	Stomach	Mucinous adenocarcinoma	3	T2N0M0	Ib
I5	56	M	Stomach	Mucinous adenocarcinoma	3	T3N0M0	II
I6	59	M	Stomach	Adenocarcinoma (sparse)	3	T1N0M0	Ia
I7	52	F	Stomach	Signet ring cell carcinoma	-	T3N0M0	II
I8	47	M	Stomach	Adenocarcinoma (sparse)	3	TisN0M0	0
I9	56	F	Stomach	Hepatoid adenocarcinoma	-	T3N0M0	II
I10	55	M	Stomach	Squamous cell carcinoma	3	T3N0M0	II
I11	16	M	Stomach	Normal stomach tissue	-	-	-

I12	50	M	Stomach	Normal stomach tissue	-	-	-
I13	25	M	Stomach	Normal stomach tissue	-	-	-
I14	35	M	Stomach	Normal stomach tissue	-	-	-
I15	30	M	Stomach	Normal stomach tissue	-	-	-
J1	66	M	Stomach	Adenocarcinoma	2	T2N0M0	Ib
J2	60	M	Stomach	Adenocarcinoma	3	T3N0M0	II
J3	56	M	Stomach	Adenocarcinoma	2	T2N0M0	Ib
J4	56	M	Stomach	Mucinous adenocarcinoma	3	T2N0M0	Ib
J5	56	M	Stomach	Mucinous adenocarcinoma	3	T3N0M0	II
J6	59	M	Stomach	Mucinous adenocarcinoma	3	T1N0M0	Ia
J7	52	F	Stomach	Signet ring cell carcinoma	-	T3N0M0	II
J8	47	M	Stomach	Signet ring cell carcinoma	-	TisN0M0	0
J9	56	F	Stomach	Hepatoid adenocarcinoma	-	T3N0M0	II
J10	55	M	Stomach	Squamous cell carcinoma	3	T3N0M0	II
J11	16	M	Stomach	Normal stomach tissue	-	-	-
J12	50	M	Stomach	Normal stomach tissue	-	-	-
J13	25	M	Stomach	Normal stomach tissue	-	-	-
J14	35	M	Stomach	Normal stomach tissue	-	-	-
J15	30	M	Stomach	Normal stomach tissue	-	-	-

Table S8: Clinical samples information from Xijing Hospital of Digestive Diseases

No.	Sex	Pathology diagnosis	TNM Stage
1	M	Stomach Adenocarcinoma	T4aN3aM0
2	M	Stomach adenocarcinoma	T3N3aM0
3	F	Stomach Adenocarcinoma	T4aN3aM0
4	M	Stomach Adenocarcinoma	T3N3aM0
5	F	Squamous cell carcinoma of the stomach	T4aN3aM0
6	M	Stomach Adenocarcinoma	T3N2M0
7	M	Stomach Adenocarcinoma	T4aN2M0
8	M	Stomach Adenocarcinoma	T4aN2M0
9	M	Stomach Adenocarcinoma	T4aN2M0
10	F	Stomach Adenocarcinoma	T4aN1M0
11	M	Stomach Adenocarcinoma	T1bN1M0
12	F	Stomach Adenocarcinoma	T4aN1M0
