# Supplementary material for: ARL4C might serve as a prognostic factor and a novel therapeutic target for gastric cancer: bioinformatics analyses and biological experiments
Source: J Cell Mol Med. 2021 Mar 16;25(8):4014–27. doi: 10.1111/jcmm.16366 (PMC8051716; doi:10.1111/jcmm.16366)
Supplement: Supplementary file 2 — Supporting information [file JCMM-25-4014-s002.docx]

**Cell culture**

The human gastric carcinoma cell lines (AGS, MKN45) were obtained from the National Infrastructure of Cell Line Resource (China). AGS was established from the resected tumor in a 54-year-old woman with untreated gastric adenocarcinoma. MKN45 was established from liver metastases in a 62-year-old Japanese woman with poorly differentiated gastric adenocarcinoma. They were maintained in RPMI-1640 medium supplemented with 10% fetal bovine serum and 1% penicillin-streptomycin solution. All cell lines were cultured at 37 °C in a humidified atmosphere containing 5% CO_2_.

**Lentivirus transduction**

The human shARL4C (NM_001282431) lentiviral was designed and constructed by GeneChem (Shanghai, China). The negative control (NC) is a scramble shRNA (TTCTCCGAACGTGTCACGT). shARL4C #1: TCAAGTTCAACGAGTTCGT, shARL4C #2: TGATCCTGAAACGCAGGAA.

To establish the shARL4C stable transduction cell lines, AGS and MKN45 (1 × 10^5^) cells were infected with concentrated lentiviral stock at ~50 multiplicity of infection at a final concentration of 0.5 μg/mL polybrene for 10 h at 37°C in 24-well plates. After transferring the cells into 25-cm^2^ flasks, a final concentration of 2 µg/mL puromycin was added to remove uninfected cells. The cell culture medium was replaced every 2–3 days with fresh and puromycin-containing RPMI-1640 medium.

**siRNA transfection**

siRNA duplexes against human ARL4C were designed and constructed by GenePharma (Shanghai, China) and the negative control (NC) is a non-targeting siRNA (Table S6). For transient transfection, after reaching approximately 70% confluence in a 6-well plate, the cells were transfected with 150 nmol of siRNA with 5 μL of Lipofectamine™ 2000 transfection reagent in 1.5 mL Gibco™ Opti-MEM™ medium for 10 h at 37 °C. The medium was replaced with fresh RPMI-1640 medium. Target genes were examined at 72 h after transfection using western blot.

**Immunofluorescence (IF)**

Cells were seeded at 5 × 10^4^ cells/well into a Millicell EZ SLIDE (Millipore, USA). The next day, cells were washed with PBS twice for 5 minutes each before being fixed with 4% paraformaldehyde (PFA) for 30 minutes, followed by washing with PBS for 15 minutes. Then, the cells were treated with 0.3% Triton X-100 in PBS for 30 minutes to induce cellular permeability, followed by washing with PBS for 15 minutes again, and finally incubated with 5% BSA for 30 minutes and incubated with primary antibodies at 4 ℃ overnight. The primary antibodies used in IF included anti-E-cadherin (at 1:200, #3195, CST, USA), anti-N-cadherin (at 1:200, #13116S, CST, USA), anti-Vimentin (at 1:200, 5741S, CST, USA), and anti-ARL4C (at 1:100, ab122025, Abcam, USA), anti-Phospho-Smad2 (Ser465/467) (at 1:100, #18338, CST, USA), anti-Phospho-Smad3 (Ser423/425) (at 1:100, #9520, CST, USA). After incubation, the cells were washed with PBS three times for 5 minutes each. Then, secondary antibodies conjugated with Alexa Fluor 594 or Alexa Fluor 488 goat anti-rabbit IgG (Invitrogen, USA) were loaded into wells at room temperature (RT) for 60 minutes in the dark conditions. Cells were washed as before, followed by DAPI (Beyotime, China) for 10 minutes. The immunostaining process ended by washing with PBS before imaging. Immunofluorescence images were harvested with a laser scanning confocal microscope (Leica, Germany).

**Cell proliferation assay**

The Cell Counting Kit-8 assay (Dojindo, Japan) was used to measure the proliferation of different cell lines according to the manufacturer’s instructions. Briefly, cells were seeded at 1000–3000 cells/well into 96-well plates in quintuplicate with 200 μl culture medium. 10 μl CCK-8 solution was added to the cells at 24 h, 48 h, 72 h, 96 h and 120 h, and the cells were incubated for 2 h at 37 ℃ in 5% CO_2_. The OD values were determined by reading the absorbance at 450 nm.

**Colony formation assay**

Cells were seeded in 6-well plates at a density of 500 cells/well (AGS) or 1000 cells/well (MKN45). After 10–14 days, cells were fixed with 4% PFA for 30 minutes and then stained with 0.05% crystal violet for 10 minutes at RT. Finally, the images of colonies were counted by ImageJ.

**RNA extraction and RT-PCR**

Total RNA of AGS and MKN45 cells was extracted by the TRIzol reagent (Takara, Japan). Then, 1000 ng RNA was reverse transcribed into cDNA by the PrimeScript™ RT Master Mix (Takara, Japan) on an iCycler iQ system (Bio-Rad, USA). RT-PCR was conducted using TB Green® Fast qPCR Mix kit (Takara, Japan) on a Light Cycler 480 (Roche, USA). The primer sequences used in our study were shown in Table S5. All reactions were performed in triplicate.

**Western blot analysis**

Cell lysates were prepared using RIPA buffer (HEART, China) including fresh protease and phosphatase inhibitors. The cells were incubated on ice for 30 minutes before centrifugation at 12,000 rpm at 4˚C for 15 minutes. Protein concentration was determined using a BCA protein assay kit (HEART, China). The protein samples were boiled in 5 × SDS protein sample buffer (Beyotime, China) for 15 minutes for complete denaturation. Protein (20–50 µg) was separated by SDS-PAGE and transferred to NC membranes (Millipore, USA). Then, the NC membranes were blocked with 5% defatted milk at RT for 2 hours and incubated with primary antibodies at 4 °C overnight. The primary antibodies used in our study included anti-ARL4C (at 1:500, ab122025, Abcam, USA), anti-E-cadherin (at 1:1000, #3195, CST, USA), anti-N-cadherin (at 1:1000, #13116S, CST, USA), anti-Vimentin (at 1:1000, #5741S, CST, USA), anti-Smad2 (at 1:1000, #5339, CST, USA), anti-Smad3 (at 1:1000, #9523, CST, USA) and anti-Phospho-Smad2 (Ser465/467) (at 1:100, #18338, CST, USA), anti-Phospho-Smad3 (Ser423/425) (at 1:100, #9520, CST, USA).The membranes were washed with TBS with Tween® 20 (TBST) three times for 10 minutes each, followed by incubation with the corresponding secondary antibodies (MYBiotech, China) at a 1:50,000 dilution for an hour at RT. The membranes were washed as before and visualized using an enhanced chemiluminescent substrate (Millipore, USA). For verification of equal protein loading, polyclonal rabbit anti-GAPDH (at 1:6000, 10494-1-AP, Proteintech Technology, UK) was used as a loading control.

**Mouse model**

Six-week-old male BALB/C nude mice were used for all experiments in this study. All animal procedures were performed according to the guidelines approved by the Animal Ethics Committee of the Fourth Military Medical University (Xi’an, China).

MKN45 cells transfected with shARL4C and negative control were injected into the subcutaneous tissue at a dose of 1 × 10^7^ cells/ml (n = 8 per group). After 4 weeks, the tumors were separated and weighed when animals were sacrificed.

For the lung metastasis assay, mice (n = 5/group) were randomly administered target cells through the tail vein (2 × 10^6^ cells in 100 μL of PBS). After 10 weeks, the mice were anesthetized and sacrificed, and histological assessments of the lungs were performed by hematoxylin-eosin (H&E) staining.

**Immunohistochemistry (IHC)**

Gastric cancer tissue microarray sections were deparaffinized by 100% xylene twice at RT for 10 minutes each. This step was followed by two incubations in 100% ethanol for 10 minutes each and rehydration ethanol with decreasing concentrations (90% and 70%; vol/vol in water, 10 minutes each) before a final 5-minute incubation in water. Antigen retrieval was used in a target retrieval citrate buffer at 95 °C for 15 minutes. The sections were cooled for 15 minutes and rinsed with PBS, and then were incubated in 3% H_2_O_2_-methanol for 5 minutes. After washed with PBS, the sections were incubated with protein blocking buffer for 10 minutes. The sections were incubated with anti-ARL4C (1:100, ab122025, Abcam, USA) overnight. After washed with PBS, the sections were incubated with a peroxidase-labeled secondary antibody for 30 minutes, and then were rinsed in PBS before reacting with liquid DAB^+^. Finally, the sections were counterstained with hematoxylin.
